# Expected effects of adopting a 9 month regimen for multidrug-resistant tuberculosis: a population modelling analysis

**DOI:** 10.1016/S2213-2600(16)30423-4

**Published:** 2017-03

**Authors:** Emily A Kendall, Anthony T Fojo, David W Dowdy

**Affiliations:** a Division of Infectious Diseases, Johns Hopkins University School of Medicine, Baltimore, MD, USA; b Division of General Internal Medicine, Johns Hopkins University School of Medicine, Baltimore, MD, USA; c Department of Epidemiology, Johns Hopkins Bloomberg School of Public Health, Baltimore, MD, USA

## Abstract

**Background:**

In May, 2016, WHO endorsed a 9 month regimen for multidrug-resistant tuberculosis that is cheaper and potentially more effective than the conventional, longer (20–24 month) therapy. We aimed to investigate the population-level implications of scaling up this new regimen.

**Methods:**

In this population modelling analysis, we developed a dynamic transmission model to simulate the introduction of this short-course regimen as an instantaneous switch in 2016. We projected the corresponding percentage reduction in the incidence of multidrug-resistant tuberculosis by 2024 compared with continued use of longer therapy. In the primary analysis in a representative southeast Asian setting, we assumed that the short-course regimen would double treatment access (through savings in resources or capacity) and achieve long-term efficacy at levels seen in preliminary cohort studies. We then did extensive sensitivity analyses to explore a range of alternative scenarios.

**Findings:**

Under the optimistic assumptions in the primary analysis, the incidence of multidrug-resistant tuberculosis in 2024 would be 3·3 (95% uncertainty range 2·2–5·6) per 100 000 population with the short-course regimen and 4·3 (2·9–7·6) per 100 000 population with continued use of longer therapy—ie, the short-course regimen could reduce incidence by 23% (10–38). Incidence would be reduced by 14% (4–28) if the new regimen affected only treatment effectiveness and by 11% (3–24) if it affected only treatment availability. Under more pessimistic assumptions, the short-course regimen would have minimal effect and even potential for harm—eg, when 30% of patients are ineligible for the new regimen because of second-line drug resistance, we projected a change in incidence of −2% (−20 to +28). The new regimen's effect was greater in settings with more ongoing transmission of multidrug-resistant tuberculosis, but results were otherwise similar across settings with different levels of tuberculosis incidence and prevalence of multidrug resistance.

**Interpretation:**

The short-course regimen has potential to substantially lessen the multidrug-resistant tuberculosis epidemic, but this effect depends on its long-term efficacy, its ability to expand treatment access, and the role of second-line drug resistance.

**Funding:**

US National Institutes of Health and Bill & Melinda Gates Foundation.

## Introduction

Multidrug-resistant tuberculosis—present in 3–4% of new tuberculosis cases and 20% of previously treated cases worldwide (with much higher prevalence in some countries)—causes 190 000 deaths each year and is a major challenge to clinicians and policy makers.^1^ Fewer than half of all notified cases with underlying multidrug resistance are identified as such, and with the scale-up of Xpert MTB/RIF, many patients diagnosed with rifampin resistance have no access to appropriate treatment. In individuals appropriately treated for multidrug-resistant tuberculosis, conventional, 20–24 month regimens (subsequently referred to as longer therapy) have a success rate of only 50% worldwide^2^ because of factors such as low drug effectiveness,^2^, ^3^ lengthy and toxic regimens that are difficult to complete,^4^ and high rates of prevalent^5^ and acquired resistance^6^ to second-line drugs. Treatment of multidrug-resistant tuberculosis is also resource intensive, costing thousands of US dollars per patient^7^ and consuming up to half of tuberculosis control budgets in high-burden countries.^1^

A potential solution to these challenges is the use of a shorter, cheaper, more effective, and more tolerable new regimen to expand treatment capacity and improve treatment success. In May, 2016, WHO made a conditional recommendation for a new short-course regimen that can treat most patients with multidrug-resistant tuberculosis in 9–12 months.^8^ This regimen consists of an initial 4–6 month phase of seven drugs including a second-line injectable, followed by a 5 month continuation of four of the oral drugs including pyrazinamide and a fluoroquinolone. It costs less than US$1000 per patient and has shown promising effectiveness, with more than 80% of patients cured in initial observational cohorts.^9^, ^10^, ^11^, ^12^ WHO now recommends this short-course regimen for patients with multidrug-resistant pulmonary tuberculosis without confirmed or probable resistance to key drugs in the regimen, while acknowledging the low capacity to test for such resistance in many settings.^13^

**Research in context**

**Evidence before this study**

Multidrug-resistant tuberculosis has a tremendous toll on patients who have to endure nearly 2 years of treatment, while exerting pressure on the budgets of tuberculosis control programmes and posing a major barrier to tuberculosis elimination worldwide. In May, 2016, WHO recommended a short-course regimen on the basis of promising individual-level effectiveness in several observational studies; however, to the best of our knowledge, the population-level implications of this recommendation have not been assessed.

**Added value of this study**

In this study, we estimated the epidemiological benefit of adopting the newly endorsed short-course regimen for multidrug-resistant tuberculosis. We also explored the extent to which the anticipated effect depends on characteristics of the regimen that remain to be determined, such as treatment success under programmatic conditions, durability of effectiveness, exclusions on the basis of additional drug resistance, treatment outcomes after such exclusions, and the extent to which cost savings from the new regimen can be used to expand treatment access. We provided a numerical estimate of the potential population-level effect of the short-course regimen in a representative setting—a 23% reduction in incidence after 8 years—and explored factors that modify this projection under different conditions. Under some reasonable sets of assumptions (eg, lower effectiveness of the short-course regimen than that suggested in initial observational studies or a higher prevalence of resistance to second-line drugs), the new regimen was projected to result in minimal, or even negative, effects on the incidence of multidrug-resistant tuberculosis.

**Implications of all the available evidence**

The new short-course regimen can potentially have an important role in the control of multidrug-resistant tuberculosis. However, this effect needs to be balanced against uncertainties related to long-term effectiveness and the importance of additional drug resistance. To optimise the effect of this new regimen, early-adopter countries should simultaneously expand diagnosis and treatment of multidrug-resistant tuberculosis and closely monitor treatment outcomes in both patients receiving the regimen and those ineligible because of additional drug resistance. An important, positive population-level effect of introducing this regimen is realistic but cannot be assumed without further evidence on the role of resistance to second-line drugs and long-term efficacy data from ongoing clinical trials.

However, unknowns about this new short-course regimen exist. First, although this regimen seemed highly effective in observational cohorts,^8^ the first rigorous comparison of its efficacy with that of longer therapy will not be completed until 2018.^14^ Second, use of this regimen necessitates testing for susceptibility to additional drugs (fluoroquinolones and second-line injectables, resistance to which is common in some populations with multidrug-resistant tuberculosis^15^). Moreover, great uncertainty exists regarding treatment outcomes in patients with resistance to other components of the regimen^8^ (particularly pyrazinamide, to which half or more multidrug-resistant strains are resistant^5^), raising concerns about the regimen's effectiveness and usefulness in geographical settings with more extensive resistance than the settings where the regimen was developed and first tested.^16^ Furthermore, whether tuberculosis programmes and health systems can truly use resources freed by a shorter regimen to expand treatment access remains uncertain. Finally, the effect of this regimen might differ substantially from one epidemiological setting to another.

In light of these uncertainties, we aimed to use a dynamic transmission model to investigate the potential effect of this new short-course regimen and to project outcomes under different assumptions regarding regimen effectiveness, treatment access, treatment outcomes in patients with additional drug resistance, and underlying epidemiology of multidrug-resistant tuberculosis.

## Methods

### Model overview

**Figure 1:**
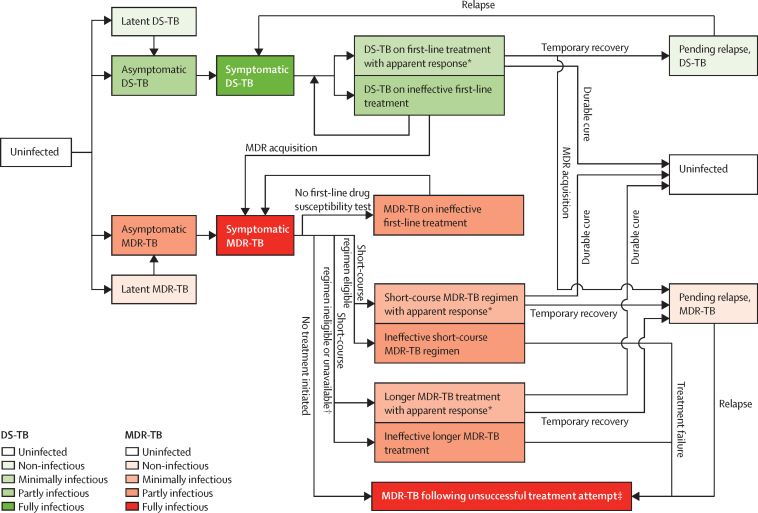
Model structure Possible movements between the main states of the model are shown. Once first-line drug susceptibility testing is performed and indicates first-line resistance, assignment to an MDR-TB treatment regimen depends on the availability of the short-course regimen and the prevalence of additional resistance as detected by the accompanying second-line drug susceptibility tests. Mortality from all compartments (higher during active tuberculosis) and stratification by tuberculosis treatment history were also modelled but not shown. DS-TB=drug-susceptible tuberculosis. MDR-TB=multidrug-resistant tuberculosis. *Individuals on treatment with apparent response have improvement in symptoms followed either by durable cure (ie, no further active disease unless reinfected) or by temporary recovery only (ie, with relapse at some point after treatment). New acquisition of MDR-TB might occur during both apparently effective and ineffective first-line treatment of DS-TB. †Patients who are suspected or documented as having additional drug resistance are ineligible for the short-course regimen—which patients fall into this category depends on the prevalence of additional resistance and the second-line drug susceptibility tests that accompany the short-course regimen. ‡Includes patients who failed MDR-TB treatment with either regimen, who relapsed after treatment with either regimen, and those with known MDR-TB who never initiated treatment because of pretreatment loss to follow-up or low capacity of tuberculosis control programmes. These individuals—like those in other active, untreated tuberculosis compartments—were modelled as having an ongoing risk of tuberculosis-related mortality and an ongoing possibility of spontaneous resolution.

In this population modelling analysis, we developed a compartmental transmission model of a multidrug-resistant tuberculosis epidemic, similar to previous tuberculosis models,^17^, ^18^ with explicit representation of diagnosis and treatment of multidrug resistance (figure 1; see appendix for description of the full model). In brief, both drug-susceptible and multidrug-resistant strains circulate in a population, with multidrug resistance emerging during treatment of drug-susceptible disease^19^ and subsequently also spreading through person-to-person transmission. Active tuberculosis, once symptomatic, is identified and treated at a given rate, but only a proportion of patients are tested for multidrug resistance and treated accordingly. Treatment is either apparently effective (ie, symptoms and infectiousness resolve, followed by lasting cure or by temporary resolution with subsequent relapse to active disease) or ineffective (ie, associated with ongoing tuberculosis mortality risk and infectiousness). Longer therapy was modelled as lasting a median of 20 months and representing a full attempt at treatment, including any changes made to the initial regimen based on clinical response or drug susceptibility testing results; outcomes were based on results in observational cohorts.^3^ We assumed that those who do not respond to a full treatment attempt for multidrug-resistant tuberculosis remain infectious until either death or spontaneous resolution.

### Calibration

To explore a large and representative number of scenarios consistent with these data, we considered 2 million sets of model parameters drawn from distributions based on the literature (table 1; appendix pp 7–8). We used log-normal distributions for continuous measures bounded from 0 to infinity, logit-normal distributions for continuous measures bounded from 0 to 1, and uniform distributions when data to suggest a most likely value were missing or sparse. In the primary analysis, we calibrated the model to a setting characterised by WHO estimates of incidence, prevalence, and mortality of tuberculosis, as well as prevalence of multidrug resistance in new and retreatment tuberculosis notifications, in people aged 15 years and older for the WHO southeast Asian region—ie, Bangladesh, Bhutan, North Korea, India, Indonesia, Maldives, Myanmar, Nepal, Sri Lanka, Thailand, and Timor-Leste—in 2014 (table 2; appendix pp 10–11).^1^

To model the expansion of diagnosis and treatment of multidrug-resistant tuberculosis in the past decade, we linearly increased the proportions of patients who are identified as having drug resistance (eg, by Xpert MTB/RIF) and considered for treatment over time, from zero in 2004 to reported levels (3·8% of new tuberculosis cases and 67% of retreatment cases) in 2014. For the primary analysis, we assumed that, in absence of a short-course regimen, the probability of receiving multidrug-resistant tuberculosis treatment would subsequently remain constant (reflecting a relatively fixed treatment budget), whereas the short-course regimen allows expansion of case detection and treatment, reflecting the lower cost and resource requirement of the new regimen.

### Modelling of short-course regimen

We modelled the introduction of a short-course regimen as an instantaneous switch from the conventional, longer therapy in 2016 for patients who are diagnosed with multidrug-resistant tuberculosis and not found to have additional drug resistance that makes them ineligible. This scenario reflects a simulated policy change with rapid restructuring of the treatment programme for multidrug-resistant tuberculosis.

To estimate the number of additional patients who could be treated in a budget-neutral introduction of the new regimen, we compared the costs of drugs and clinical care for each regimen. Drug costs for the short-course regimen are less than half of those of longer therapy, and the shortened durations of the intensive phase and the overall treatment course also reduce other associated health-care costs,^8^ whereas added costs of second-line drug susceptibility testing are small relative to the total cost of treatment.^26^ For simplicity, we assumed in the primary analysis that introduction of the short-course regimen would allow twice as many patients to be treated on the same multidrug-resistant tuberculosis treatment budget. We implemented this doubling by expanding the number of patients with multidrug-resistant tuberculosis offered treatment, first to previously treated patients and then to new patients.

In the primary analysis, we modelled a scenario in which roughly 10% of patients with multidrug-resistant tuberculosis have additional drug resistance (or suspected resistance) that disqualifies them from the short-course regimen, leading to very poor outcomes. We used a median duration of 10 months for the short-course regimen and 20 months for longer therapy,^9^ and assumed that loss to follow-up is reduced by half with the short-course regimen. Treatment success for people remaining in treatment was set at 92·5% for the short-course regimen^9^ and 66–85% for longer therapy;^3^ these percentages include only those who are not lost to follow-up and are therefore higher than reported figures that do not distinguish between loss to follow-up and other adverse outcomes. Relapse risk after successful treatment was set at 1% for the short-course regimen and 1–10% for longer therapy. The estimated outcomes of the short-course regimen were based on results from an observational cohort study in Bangladesh;^9^ similar results were obtained elsewhere.^8^, ^12^ The estimated 10% ineligibility for the short-course regimen is based on the assumptions that patients would be screened for second-line drug resistance with a line probe assay (of imperfect sensitivity),^27^ moxifloxacin resistance would be similar to levels observed in Pakistan and Bangladesh,^5^ and monoresistance to second-line injectables would be rare.^28^, ^29^ We also assumed, conservatively, that patients found to have such disqualifying additional drug resistance would have very poor outcomes, comparable to those reported for extensively drug-resistant tuberculosis^1^ and to tuberculosis outcomes in the pre-antibiotic era^30^ (ie, that half of these patients will ultimately die of tuberculosis, although such deaths might occur well after treatment is completed).

We explored several alternative scenarios to the above assumptions (table 3). Alternatives involving inter-related aspects of prevalence, diagnosis, and associated treatment outcomes of second-line drug resistance were explored combinatorially (table 4).

The primary outcome for each scenario was the percentage reduction in multidrug-resistant tuberculosis incidence in 2024, compared with projections under continued use of longer therapy. Results are reported as the median simulated value and corresponding 95% uncertainty range (UR), reflecting the 2·5th to 97·5th percentile of data-consistent simulations.

### Sensitivity analyses

We assessed the sensitivity of the primary results to the value of all underlying model parameters. We also assessed the sensitivity of our results to assumptions about ongoing scale-up of drug susceptibility testing even in the absence of the short-course regimen, to underlying dynamics of acquisition, transmission, and reactivation of multidrug-resistant tuberculosis, and to alternative epidemiological scenarios reflecting a range of tuberculosis incidence and multidrug-resistant tuberculosis prevalence (appendix).

### Role of the funding source

The funder of the study had no role in study design, data collection, data analysis, data interpretation, or writing of the report. The corresponding author had full access to all the data in the study and had final responsibility for the decision to submit for publication.

## Results

Our model generated 11 289 data-consistent simulations, which fitted well with our epidemiological calibration targets (table 2). Posterior distributions of model parameters favoured lower rates of acquisition and transmission of multidrug-resistant tuberculosis (reflecting that multidrug resistance is present in only 2% of new tuberculosis notifications, despite decades of treatment with isoniazid and rifampin) but otherwise suggested no strong support for specific parameter values within the ranges of the specified prior distributions (appendix p 13).

**Figure 2:**
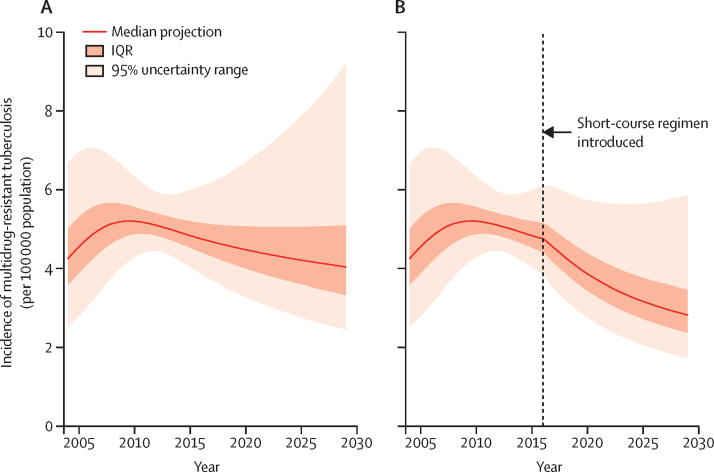
Projected incidence of multidrug-resistant tuberculosis in the primary scenario (A) Continued use of longer therapy. (B) Implementation of the short-course regimen in 2016.

Assuming that current practices continue, we projected that the incidence of multidrug-resistant tuberculosis would decrease by a median of 14% (95% UR −36 to 39) from 4·9 [95% UR 4·2–5·9] per 100 000 population in 2014 to 4·3 [2·9–7·6] per 100 000 population in 2024 (figure 2A), reflecting higher levels of treatment than in the past. However, the large 95% UR reflects the paucity of longitudinal data. Despite this uncertainty in the overall trajectory of multidrug-resistant tuberculosis incidence, the short-course regimen was consistently projected to have benefit under the assumptions of the primary scenario. We projected that, 8 years after introduction of the short-course regimen, the incidence of multidrug-resistant tuberculosis would be 3·3 (2·2–5·6) per 100 000 population—ie, the incidence in 2024 would be 23% (10–38) lower with the short-course regimen (figure 2B). A slightly larger reduction in multidrug-resistant tuberculosis mortality (31%, 14–46) was projected than for incidence.

**Figure 3:**
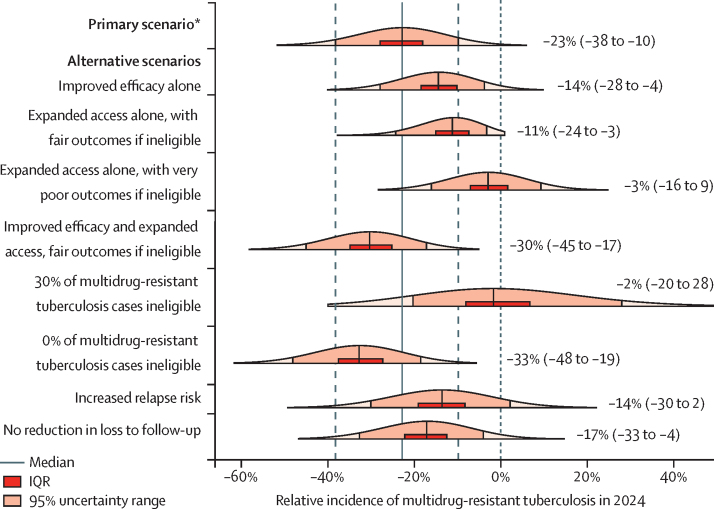
Projected change in incidence of multidrug-resistant tuberculosis in 2024 under the short-course regimen relative to longer therapy, by scenario Median (95% uncertainty range) is shown beside each plot; the height of each plot corresponds to the probability density of the model projections. See table 3 for further descriptions of each scenario. *Improved long-term efficacy, doubling of treatment access, very poor outcomes for the 10% of patients with multidrug-tuberculosis who are ineligible for the short-course regimen, halving of losses to follow-up, and reduced relapse risk.

**Figure 4:**
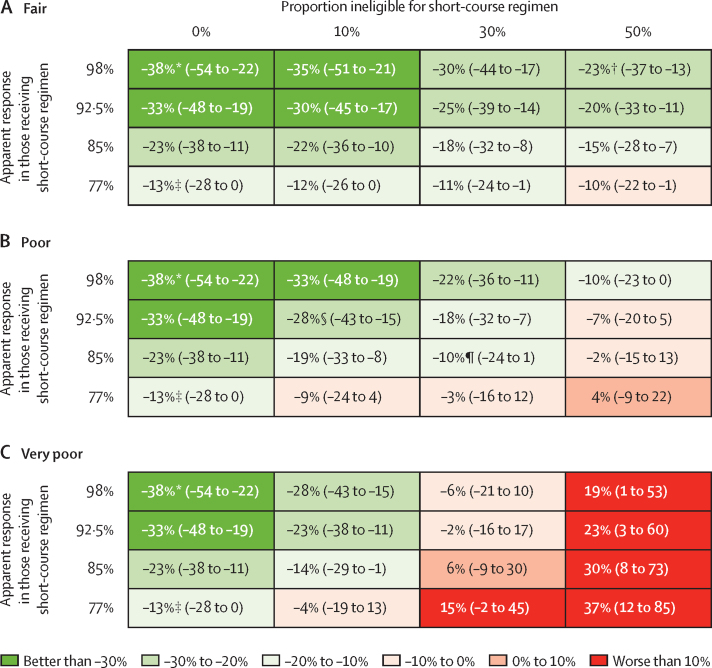
Relative change in incidence of multidrug-resistant tuberculosis in 2024, under different combinations of apparent response to short-course regimen and regimen exclusions, by treatment outcome in those excluded (A) Fair—77% apparent response, with 4% relapse. (B) Poor—50% durably cured at end of therapy. (C) Very poor—20% cured at 2 years. Data are median (95% uncertainty range). Differences in modelled treatment outcomes reflect not only the efficacy of the regimen but also the prevalence of additional drug resistance in the population and the drug susceptibility test used. Since these underlying values are difficult to measure, this figure provides decision makers with projections of impact according to three measurable parameters. The rationale for these characteristics is explained in table 4. Specific scenarios of interest, which assume published point estimates of efficacy of the short-course regimen in different subgroups,^8^ are indicated as follows. *Second-line line probe assay screening in an area of very low prevalence of additional resistance. †Full phenotypic drug susceptibility tests (or full rapid drug susceptibility tests if available in the future) used in an area of moderate prevalence of second-line resistance. ‡Regimen implemented without having second-line drug susceptibility tests available in an area of moderate prevalence of second-line resistance. §Second-line line probe assay screening in an area of moderate prevalence of additional resistance. ¶Second-line line probe assay screening in an area of high prevalence of additional resistance.

The magnitude of the short-course regimen's effect on the incidence of multidrug-resistant tuberculosis was dependent on several key assumptions (Figure 3, Figure 4). If the short-course regimen only improved outcomes in patients treated but did not facilitate treatment access (alternative scenario 1), it was projected to reduce incidence by only 14% (95% UR 4–28). Similarly, if the short-course regimen's benefit was restricted to expansion of treatment access alone and did not change the average treatment outcome (alternative scenario 2), then the incidence in 2024 was projected to fall by 11% (3–24). Furthermore, if we assumed that a finding of equivalent efficacy between the two regimens was dependent on the short-course regimen only being used in those without additional resistance—while those excluded from the short-course regimen had very poor outcomes (alternative scenario 3)—then the short-course regimen could have a minimal effect on the multidrug-resistant tuberculosis epidemic as a whole (relative change in incidence −3%, −16 to 9), despite doubling the number of people treated. Similarly pessimistic projections were seen when the prevalence of disqualifying drug resistance was increased to 30% (alternative scenario 5). Figure 4 shows the projected effects of the short-course regimen in a given setting as a function of three measurable parameters: treatment outcomes in those who take the short-course regimen; treatment outcomes in those excluded from the regimen (and instead given longer therapy); and the proportion of the population excluded from the short-course regimen.

In sensitivity analyses, the relative effect of the short-course regimen did not depend substantially on the degree of future scale-up of drug susceptibility testing (appendix p 14). Other variables that strongly influenced the effect of the short-course regimen included the long-term efficacy of longer therapy and assumptions about the duration and trajectory of the multidrug-resistant tuberculosis epidemic (appendix pp 15–16). The regimen's effect showed little sensitivity to the balance of acquired versus transmitted multidrug resistance and was only moderately sensitive to the balance of recent versus remote transmission (appendix p 17). Similarly, the short-course regimen had a greater potential effect in high-prevalence settings; results were otherwise similar across a range of simulated epidemiological settings (appendix p 18).

## Discussion

This epidemic model suggests that implementation of the short-course regimen could have an important effect on the multidrug-resistant tuberculosis epidemic, with an estimated 23% reduction in incidence over 8 years relative to continued use of longer therapy. This effect depends on key assumptions, including improved long-term effectiveness, the ability to use resource savings to expand access, and minimised poor outcomes resulting from additional drug resistance. If these assumptions prove incorrect, then the short-course regimen could have minimal or even detrimental effect—eg, possibly having no effect on the incidence of multidrug-resistant tuberculosis even if the number of people treated could be doubled. These findings emphasise the need for additional data collection as the short-course regimen is rolled out and highlight that implementation of this regimen could have important population-level effects, but also that this result is far from certain.

More effective regimens for multidrug-resistant tuberculosis are sorely needed, and a substantial proportion of the projected impact of a shorter regimen derives from the assumption of superior efficacy in those treated. The high treatment success rates (>80%) and low relapse risks (<1%)^8^ of the short-course regimen observed in initial cohorts are promising compared with longer therapy (50% success rate worldwide^1^ and 62% in those who would have met inclusion criteria for the short-course regimen).^8^ Whether efficacy of this new regimen is truly superior (and durable) awaits the results of an ongoing clinical trial.^14^ Our results suggest that if the short-course regimen is not more efficacious than longer therapy in eligible patients, then its impact will largely depend on whether it can facilitate expansion of treatment access and whether patients with disqualifying resistance can be appropriately triaged and successfully treated. In hotspots of more extensive drug resistance, the conditions under which the short-course regimen offers benefit will be more limited and will depend even more on the achievable gains in efficacy and resource use.

Because of the high cost of traditional care, the potential to diagnose and treat more patients within constrained budgets contributes strongly to the short-course regimen's potential effects. Our projections are similar to an estimate of the effect of universal Xpert use in India, accompanied by gradual improvement in treatment outcomes (ie, 25% reduction in incidence of multidrug-resistant tuberculosis over a decade).^31^ However, unlike that analysis, we explored a mechanism (short-course regimen) by which such increased treatment access and improved treatment outcomes could potentially be achieved in a budget-neutral manner, if per-patient savings were used to identify and treat more patients. If resources were reallocated elsewhere, the effect of the short-course regimen on incidence would shrink, but the overall impact on burdened tuberculosis control programmes and health systems, as well as on patients for whom multidrug-resistant tuberculosis can be economically devastating, could remain substantial. Future analyses to explicitly assess the economic effects of the short-course regimen are warranted. We also assumed that availability of clofazimine will meet demands, that second-line drug susceptibility can be tested before patients are lost to follow-up, and that the short-course regimen will be scaled up rapidly. To the extent that scale-up is slow, incomplete, or associated with increased pretreatment losses to follow-up, the effect will be diminished. Moreover, although children and extrapulmonary tuberculosis contribute little to tuberculosis transmission, the still-uncertain usefulness of the short-course regimen in such populations will affect its ability to reduce morbidity and mortality of multidrug-resistant tuberculosis.

Our model highlights an important drawback of the short-course regimen: its reliance on component drugs to which resistance is prevalent in some populations.^5^, ^32^ At baseline, we assumed that 10% of people without previous treatment for multidrug-resistant tuberculosis would be identified as having resistance to fluoroquinolones or second-generation aminoglycosides (ie, contraindications to the short-course regimen) and excluded on that basis. In settings where this proportion is 30%^33^ or higher,^34^ we projected a substantially diminished effect of the new regimen. Similarly, settings that implement the short-course regimen without sufficient capacity for rapid second-line drug susceptibility testing might experience reduced effectiveness and diminished short-term benefit, as well as long-term risk of amplified second-line drug resistance. Pyrazinamide resistance also could limit the effect of the short-course regimen. Pyrazinamide is included for the whole duration of the regimen and might be important for ensuring good treatment outcomes or preventing additional drug resistance, but 37–81% of multidrug-resistant strains might be pyrazinamide resistant.^5^ Therefore, assessment of pyrazinamide's role is urgently needed; if further study determines that individuals with resistance to pyrazinamide should also be excluded from this regimen (resulting in exclusion of nearly 50% of patients with multidrug-resistant tuberculosis in southeast Asia^5^ and a greater proportion in some other settings^35^), then the regimen's population-level effect is likely to be very small.

As with all modelling studies, our analysis has some limitations. Our model projections reflect uncertainty related to trends in resistance to first-line and second-line drugs, rapidly changing diagnostic and treatment practices, and the scarcity of data on the population dynamics of multidrug-resistant tuberculosis. Importantly, our homogeneously mixed model could have overestimated the effect of this regimen in specific settings. We also simplified the dynamic representation of drug resistance to only two strains. Resistance to other drugs was implicitly factored into treatment outcomes, but transmission of multiple drug-resistant strains was not explicitly modelled. For this reason, we limited projections to a relatively short (<10 year) timeframe over which the selection of resistance to second-line drugs is expected to have relatively little epidemiological effect. Mounting second-line resistance, if it occurs, could lead to worse outcomes over time than those projected here, especially in the long term. Future modelling analyses could assess the effect of the short-course regimen on the acquisition and emergence of fluoroquinolone resistance. We were also unable to model all the complexities of tuberculosis epidemics—for example, we did not explicitly model individual heterogeneity in HIV or diabetes status or variation in tuberculosis-associated or background mortality over time, but these factors might be important considerations in certain settings.

In summary, this modelling analysis illustrates the potential important effects of a newly recommended short-course regimen on the multidrug-resistant tuberculosis epidemic. However, it also highlights that this effect is dependent on certain key factors, including the regimen's long-term efficacy, the ability to facilitate scale-up of treatment access through resource savings, and the number and outcomes of patients who are excluded on the basis of additional drug resistance. Crucial data in estimating the ultimate effect of this regimen include evidence of durable efficacy from randomised controlled trials and data for the effect of pyrazinamide resistance, which is highly prevalent in patients with multidrug resistance. Additional research to develop improved regimens in the future will be essential, in view of the key limitations of the present short-course regimen. Ultimately, in making urgent decisions about whether to implement this new regimen at the country and global levels, the potential to reduce incidence by 20% or more needs to be weighed against the substantial uncertainty still surrounding the long-term effects of this regimen on the population dynamics of multidrug-resistant tuberculosis.

**This online publication has been corrected. The corrected version first appeared at thelancet.com/respiratory on Jan 5, 2017**

## Figures and Tables

**Table 1:** Select model parameters

		**Median estimate**	**Distribution**	Sampling range*
Probability of rapid progression after initial tuberculosis infection^20^		0·14	Logit-normal	0·08–0·25
Protection against rapid progression after reinfection, if latently infected^21^		0·5	Logit-normal	0·1–0·9
Reactivation rate from latent to early (asymptomatic) active tuberculosis,^22^ per year		0·001	Log-normal	0·0005–0·002
Rate of tuberculosis diagnosis and treatment initiation,^1^ per year		1	Log-normal	0·7–1·5
Proportion failing to initiate treatment for multidrug-resistant tuberculosis after diagnosis (in excess of loss to follow-up of patients with drug-susceptible tuberculosis)^1^		0·05	Logit-normal	0·03–0·10
Proportion of treated patients who have an apparent treatment response†				
	Newly diagnosed patients with drug-susceptible tuberculosis, first-line therapy^1^	0·98	Logit-normal	0·96–0·99
	Patients with multidrug-resistant tuberculosis, longer therapy^3^	0·77	Logit-normal	0·66–0·85
Proportion who relapse, among those with apparent treatment response				
	Newly diagnosed patients with drug-susceptible tuberculosis, first-line therapy^19^	0·040	Logit-normal	0·026–0·060
	Patients with multidrug-resistant tuberculosis, longer therapy^23^	0·040	Logit-normal	0·015–0·100
Probability of loss to follow-up during therapy				
	First-line therapy^1^	0·06	Logit-normal	0·03–0·1
	Longer therapy for multidrug-resistant tuberculosis^1^	0·19	Logit-normal	0·14–0·25
Relative transmissibility of multidrug-resistant strain^24^		0·60	Log-normal	0·38–0·94
Risk of acquiring multidrug resistance during first-line therapy^19^		0·005	Logit-normal	0·0025–0·01
Proportion of patients with multidrug resistance disqualified from the short-course regimen^5^, ^25^		0·1	Logit-normal	0·07–0·15

See the appendix pp 7–8 for a complete list of parameters and additional references, and p 9 for an illustration of how the values of treatment-related parameters translate to observed treatment outcomes.

* 2·5th to 97·5th percentiles of unbounded distributions.

† Including those who might later be lost to follow-up or relapse, or both.

**Table 2:** Calibration targets and model fit

	Reported values for southeast Asia*	**Median model values (95% uncertainty range)**
Tuberculosis incidence per 100 000 adult population per year	203 (192–232)	203 (191–207)
Annual change in incidence	–2%	–2·2% (1·8–2·8)
Tuberculosis prevalence per 100 000 adult population	275 (224–330)	271 (228–323)
Tuberculosis mortality per 100 000 adult population per year	26·2 (20·9–32·6)	26·7 (21·2–32·3)
Proportion of new notifications with multidrug resistance	2·2% (1·9–2·6)	2·1% (1·9–2·5)
Proportion of retreatment notifications with multidrug resistance	16% (14–18)	16·7% (14·4–17·9)

* We derived these estimates from WHO-reported point estimates (uncertainty intervals),^1^ adjusted to reflect the burden of pulmonary tuberculosis in the adult population (ie, those aged 15 years and older), on the basis of the proportion of cases that are pulmonary, the proportion estimated to occur in those aged 15 years and older, and the proportion of the southeast Asian population aged 15 years and older.

**Table 3:** Assumptions in primary scenario and alternative scenarios, by variable

	**Primary scenario**		**Alternative scenarios**
	Baseline	Short-course regimen	
Level of treatment initiation for multidrug-resistant tuberculosis	Maintain existing levels	Double existing levels	Scenario 1—maintain existing levels in baseline and with short-course regimen; Gradual increase in baseline, doubled with short-course regimen (appendix); Gradual increase in baseline and with short-course regimen (appendix); or Immediate optimisation of drug susceptibility testing in baseline and with short-course regimen (appendix)
Proportion of patients with apparent treatment response, among those who receive the short-course regimen*	Not applicable	92·5%	Scenario 2—same as longer therapy (roughly 77%†) Scenario 3—same as longer therapy (roughly 77%†), combined with improved “fair” outcomes for those ineligible (see below)
Outcome of longer therapy for patients ineligible for the short-course regimen*	Not applicable	20% cured at end of therapy (“very poor”)‡	Scenario 4—same apparent response (roughly 77%†) as the average patient with multidrug-resistant tuberculosis in the baseline scenario (“fair”) Scenario 3—”fair” response as in Scenario 4, combined with reduced response in those who receive the short-course regimen (see above)
Proportion of patients ineligible for short-course regimen on the basis of second-line resistance and drug susceptibility testing practices*	Not applicable	10%	Scenario 5—30% Scenario 6—0%
Relapse risk in those with apparent treatment response who finish treatment course	Roughly 4% for longer therapy†	1% for short-course regimen	Scenario 7—roughly 8% (twice that of longer therapy) for short-course regimen
Loss to follow-up	Roughly 19% for longer therapy†	10% for short-course regimen	Scenario 8—roughly 19% for both regimens†

* See table 2 for further details.

† Sampled from distributions shown in table 1.

‡ Apparent treatment response not explicitly modelled; see appendix p 9 for further details of calculation.

**Table 4:** Values used in combinatorial analyses, by variable

	**Rationale**
**Proportion of patients receiving short-course regimen with apparent treatment response**	
98%, with 1% relapse	As reported with short-course regimen for patients susceptible to both fluoroquinolone and pyrazinamide^8^
92·5%, with 1% relapse	Average across all patients with multidrug-resistant tuberculosis receiving the short-course regimen in Bangladesh^9^
85%, with 2·5% relapse	As reported with short-course regimen (with large uncertainty) for patients resistant to pyrazinamide only^8^
77%, with 4% relapse	Average outcomes of longer therapy
**Outcome of longer therapy for patients ineligible for short-course regimen**	
Fair: 77% apparent response, with 4% relapse	Outcomes equivalent to the average outcome of longer therapy in the baseline scenario; might reflect effective individualisation of treatment or limited drug resistance in ineligible patients (eg, resistance to pyrazinamide only)
Poor: 50% durably cured at end of therapy*	Corresponds to outcomes of longer therapy for patients with multidrug resistance and fluoroquinolone resistance^8^
Very poor: 20% cured at 2 years†	Most conservative assumption; those excluded have typical outcomes of extensively drug-resistant tuberculosis on longer therapy
**Proportion of patients ineligible for short-course regimen on the basis of second-line resistance and drug susceptibility testing practices**	
0%	No second-line drug susceptibility testing or no second-line resistance
10%	Line probe assay (for fluoroquinolones and second-line injectables); levels of resistance similar to Pakistan and Bangladesh
30%	Line probe assay; levels of resistance similar to eastern Europe
50%	Exclusion of all patients with pyrazinamide or second-line drug resistance in a typical setting

* Corresponds to two-thirds having “fair” outcomes and a third having “very poor” outcomes.

† Modelled as an ongoing probability of roughly 13% per year of cure and an ongoing tuberculosis mortality risk, resulting in roughly 23% death, 20% cure, and 57% persisting with active disease at 2 years.
