# Corrections

**DOI:** 10.1016/S2213-2600(17)30001-2

**Published:** 2017-02

*Kendall EA, Fojo AT, Dowdy DW. Expected effects of adopting a 9 month regimen for multidrug-resistant tuberculosis: a population modelling analysis.* Lancet Respir Med 2016; published online Dec 15. http://dx.doi.org/10.1016/S2213-2600(16)30423-4—In this Article, the Open Access license has been changed to CC BY. This correction was made to the online version as of Jan 5, 2017, and the printed version will be corrected.

