# Supplementary material for: Expected effects of adopting a 9 month regimen for multidrug-resistant tuberculosis: a population modelling analysis
Source: Lancet Respir Med. 2017 Mar;5(3):191–9. doi: 10.1016/S2213-2600(16)30423-4 (PMC5332590; doi:10.1016/S2213-2600(16)30423-4)
Supplement: Supplementary appendix [file mmc1.pdf]

THE LANCET
Respiratory Medicine

Supplementary appendix

This appendix formed part of the original submission and has been peer reviewed. We post it as supplied by the authors.

Supplement to: Kendall EA, Fojo AT, Dowdy DW. Expected effects of adopting a 9 month regimen for multidrug-resistant tuberculosis: a population modelling analysis. *Lancet Respir Med* 2016; published online Dec 15. http://dx.doi.org/10.1016/S2213-2600(16)30423-4.

Supplement:
Expected impact of adopting a 9-month regimen for multidrug-resistant tuberculosis:
A population model

Model Equations

Disease states

(These denote the fraction of the population in a given state or set of states)

S = susceptible (never-infected, or cured/recovered)
L = latently infected
E = Asymptomatic, early-active TB (not yet seeking care)
A = Symptomatic active TB
B_{1e} = on apparently-effective first-line (DS-TB) treatment (leading to culture conversion and ultimately either durable cure or relapse)
B_{1i} = on ineffective first-line treatment (remain infectious)
B_{2e1} = on initial six months of apparently-effective longer MDR-TB therapy
B_{2e2} = on continuation phase of apparently-effective longer MDR-TB therapy
B_{2i} = on ineffective longer MDR-TB therapy
$B_{2e1'}$ = on initial six months of apparently-effective short-course MDR-TB therapy
$B_{2e2'}$ = on continuation phase of apparently-effective short-course MDR-TB therapy
$B_{2i'}$ = on ineffective short-course MDR-TB therapy
C = active MDR-TB after unsuccessful treatment attempt, not being further treated
W = pending relapse; no history of MDR-TB treatment
V = pending relapse; has history of previous MDR-TB treatment

X = any of the above states
I = any infectious state

Subscripts applied to the above, to denote more specific states:

Treatment status: $T = N$ (new) or P (previously diagnosed with and/or treated for TB)
Drug resistance: $D = S$ (drug-susceptible) or R (drug-resistant)
(Note: When T or D is used as index in a sum, $\bar{T}$ and $\bar{D}$ denote the opposite treatment status or drug resistance state from the one being indexed.)

Parameters:

β_0 = transmission coefficient in year 1999 (chosen to give desired year 1999 incidence when model is run to equilibrium)
f_R = transmission fitness of MDR strain, relative to $f_S = 1$ for DS strain, in year 1999
μ = baseline mortality rate of adults age > 15 years without TB
μ_{tb} = excess mortality rate of active untreated TB
i_X = infectiousness and mortality of state X, relative to active untreated TB (deparately defined for $X = E$, for $X \in \{B_{1i}, B_{2i}, B_{2i'}\}$, and for $X \in \{B_{2e1}, B_{2e1'}\}$)
ν = rate of spontaneous resolution of active TB
τ_{t1} = duration of first-line therapy (set at 6 months)
τ_{t21} = duration of MDR-TB therapy, first phase (also set at 6 months; failures, losses to follow up, or complete resolution of infectiousness are modeled as occurring after this time period, for both MDR-TB regimens)
τ_{t22}, τ'_{t22} = remaining duration of MDR-TB therapy, for longer and short-course regimens respectively

$\sigma_{N,P}$ = first-line treatment efficacy (fraction of [new, previously-treated] DS-TB patients with apparent treatment response; includes those who may experience loss to follow up and/or relapse with or without acquired resistance)

σ_2, σ'_2 = MDR-TB treatment efficacy (fraction of MDR-TB patients with apparent treatment response; includes those who may experience loss to follow up and/or relapse) for longer therapy and short-course regimen, respectively

δ_1 = fraction of patients lost to follow up during first-line therapy

δ_2, δ'_2 = fraction of patients lost to follow up during MDR-TB therapy, conventional and short-course regimens respectively

γ = Fraction of patients immediately retreated (with or without DST) after failing DS-TB therapy

$\alpha_{N,P}$ = fraction of [new, previously-treated] DS-TB patients who acquire drug resistance during therapy

$\omega_{1N,P}$ = risk of relapse after finishing apparently-effective therapy for [new, previously-treated] DS-TB patients

ω_2, ω'_2 = risk of relapse after after finishing apparently-effective MDR-TB therapy, for conventional and short-course regimens, respectively

τ_ω = mean time to relapse, among patients who will relapse

$\eta_{\delta 1}$ = fraction of DS-TB patients who return to active disease if lost to follow up during apparently-effective DS-TB therapy

$\eta_{\delta 2}$ = fraction of MDR-TB patients who return to active disease if lost to follow up during apparently-effective MDR-TB therapy

ρ = probability of rapid progression to early-active disease after infection

λ = reduction in probability of rapid progression after superinfection, if already latently infected

r = rate of reactivation (latent to early-active)

a = duration of early-active TB

x = time to TB diagnosis and treatment (incorporates overall pretreatment loss to follow up)

$s_{N,P}$ = fraction of [new, previously-treated] TB patients with MDR-TB who receive DST

b = added probability of pretreatment loss to follow up associated with an MDR-TB diagnosis

j = probability of disqualifying drug resistance, among new MDR-TB patients who are otherwise eligible for the short-course regimen

c = availability of the short course regimen ($c = 1$ when the short-course regimen has replaced conventional therapy for eligible MDR-TB patients)

z = indicator for whether those excluded from the short-course regimen maintain response rates of σ_2: $z = 0$ if conventional-therapy outcomes are maintained, or $z = 1$ if excluded individuals are assumed to have poor outcomes.

Time-dependent quantities:

t = time in years from year 1999. Evaluation of model calibration therefore occurs at $t = 15$ and short-course regimen introduction occurs at $t = 17$.

d = rate of linear decline in transmission per DS-TB case β_S, derived by fitting linear model to 2%/year decline in DS-TB incidence in 1999 and 2014

e = relative rate of decline in transmission per MDR-TB case, as fraction of d

Transmission coefficient:

$\beta_S(t) = \beta_0 - d \cdot t$
Observed declines in TB incidence are modeled as a linear decline over time in the transmission coefficient. (Sensitivity analyses consider an alternative model in which the decline in incidence is due to decreased reactivation from latency.)

$\beta_R(t) = (\beta_0 - ed \cdot t) f_R$
Transmissibility of the drug-resistant strain may be less than that of the drug susceptible strain, and transmission of this strain also decreases over time in the primary analysis.

Note: For new individuals entering the population at age 15, the fraction with latent TB is dependent on the average transmission coefficient over the preceding 15 years, or $\beta_D(t - \frac{15}{2})$ during the analysis period.

Force of infection:

$FOI_D(t) = \Sigma_{X \in I_D}(X(t) i_X \beta_D(t))$

At any given point in time, all individuals in infectious states contribute additively to the force of infection. When their infectious state is less infectious than fully active, untreated TB, their contribution to the force of infection is reduced by factor i_X.

Differential equations:

Susceptible states

$$\frac{dS_N}{dt} = \left[\sum_X (\mu + i_X \mu_{tb}) X(t)\right] e^{-\sum_D \sum_{X \in I_D} i_X X(t) \beta_D (t - \frac{15}{2})} + \nu(\sum_D (E_{ND}(t) + A_{ND}(t))) - (\mu + \sum_D FOI_D(t)) S_N(t) \quad (1)$$

Susceptibles (uninfected) with no history of prior TB treatment are generated by replacement of natural and TB-related deaths with new 15 year olds; the fraction of these 15 year olds who are susceptible rather than latently infected is based on the force of infection over the previous 15 years.
Spontaneous resolution of early-active and active TB prior to starting treatment also results in return to the never-treated susceptible compartment. Losses from this compartment occur due to natural death and due to new TB infection.

$$\begin{aligned}\frac{dS_P}{dt} &= \nu \left(\sum_D (E_{PD}(t) + A_{PD}(t)) + C_R(t)\right) \\ &+ \sum_T B_{1e(TS)}(t) \left[(1 - \alpha_T)((1 - \delta_1)(1 - \omega_{1T}) + \delta_1 (1 - \eta_{\delta 1}))/\tau_{t1}\right] \\ &+ B_{2e1}(t) \delta_2 (1 - \eta_{\delta 2})/\tau_{t21} + B_{2e1'}(t) \delta_{2'} (1 - \eta_{\delta 2})/\tau_{t21} \\ &+ B_{2e2}(t)(1 - \omega_2)/\tau_{t22} + B_{2e2'}(t)(1 - \omega_2')/\tau_{t22}' - (\mu + \sum_D FOI_D(t)) S_P(t)\end{aligned} \quad (2)$$

Spontaneous resolution of TB at rate ν in previously-treated patients with recurrent early-active, active, or chronic TB places them in the susceptible, previously-treated (i.e. cured) compartment.
Return to this compartment can also result from treatment-related cure, in DS-TB patients on effective therapy who do not experience acquired resistance, relapse, or loss to follow up, and in MDR-TB patients who are lost to follow up (modeled as occurring after the first phase of treatment) but nevertheless cured and in those who complete full MDR-TB treatment and will not relapse. Losses from this susceptible compartment can occur due to death or new infection.

Latently infected states

$$\begin{aligned}\frac{dL_{ND}}{dt} &= \left[\sum_X (\mu + i_x \mu_{tb}) X(t)\right] \frac{\sum_{X \in I_D} i_X X(t) \beta_D (t - \frac{15}{2})}{\sum_D \sum_{X \in I_D} i_X X(t) \beta_D (t - \frac{15}{2})} \left(1 - e^{-\sum_D \sum_{X \in I_D} i_X X(t) \beta_D (t - \frac{15}{2})}\right) \\ &+ (1 - \rho) FOI_D(t) S_N(t) \\ &+ (1 - (1 - \lambda)\rho) \left[\frac{f_D}{\sum_D f_D} FOI_D(t) L_{N\bar{D}}(t) - \frac{f_{\bar{D}}}{\sum_D f_D} FOI_{\bar{D}}(t) L_{ND}(t)\right] \\ &- (1 - \lambda)\rho FOI_D(t) \sum_D L_{ND}(t) - (r + \mu) L_{ND}(t)\end{aligned} \quad (3)$$

$$\begin{aligned}\frac{dL_{PD}}{dt} &= (1 - \rho) FOI_D(t) S_P(t) \\ &+ (1 - (1 - \lambda)\rho) \left[\frac{f_D}{\sum_D f_D} FOI_D(t) L_{P\bar{D}}(t) - \frac{f_{\bar{D}}}{\sum_D f_D} FOI_{\bar{D}}(t) L_{PD}(t)\right] \\ &- (1 - \lambda)\rho FOI_D(t) \sum_D L_{PD}(t) - (r + \mu) L_{PD}(t)\end{aligned} \quad (4)$$

As new 15 year olds replace natural and TB-related deaths, the fraction who enter the population with latent infection is determined by an approximation of cumulative exposure to each strain over the past 15 years; these have no history of TB treatment and are therefore added to the L_N states only).
Both new and previously-treated latent infections also arise from infection of susceptibles who do not progress rapidly (probability $1 - \rho$) and from superinfection of latently-infected individuals with a different strain (with the probability ρ of rapid progression reduced by factor $1 - \lambda$, and with the strains competing based on fitness to become the new latently-infecting strain); these latter superinfections also result in a loss from the corresponding latent compartment for the other strain ($\bar{D}$). Losses from the latently-infected compartments also occur due to super-infection with rapid progression (with probability $(1 - \lambda) * \rho$), from reactivation (rate r), and from natural mortality (rate μ).

Asymptomatic (early active) disease

$$\frac{dE_{TD}}{dt} = \rho S_T(t)FOI_D(t) + rL_{TD} + (1-\lambda)\rho FOI_D(t)\sum_D L_{TD}(t) - (1/a + \nu + \mu + i_E\mu_{tb})E_{TD}(t) \tag{5}$$

Early active disease develops after initial infection with rapid progression, after reactivation from latency, or after superinfection of latently-infected individuals with rapid progression of the new strain. Losses from this compartment occur from further progression to fully-active TB, from spontaneous resolution of early-active TB, and from natural or TB-related mortality.

Symptomatic active disease

$$\frac{dA_{NS}}{dt} = aE_{NS}(t) - (1/x + \nu + \mu + \mu_{tb})A_{NS}(t) \tag{6}$$

$$\frac{dA_{NR}}{dt} = aE_{NR}(t) - (1/x + \nu + \mu + \mu_{tb})A_{NR}(t) \tag{7}$$

For all strains and all treatment histories, active disease develops via progression from early active disease (E), and losses from these active disease compartments occur due to TB diagnosis, spontaneous resolution, and natural and TB-related death.

$$\begin{aligned}\frac{dA_{PS}}{dt} &= aE_{PS}(t) + (1/\tau_\omega)W_S(t) + \frac{1}{\tau_{t1}}\sum_T(1-\alpha_T)\left(\delta_1\eta_{\delta_1}B_{1eTS}(t) + (\delta_1 + (1-\delta_1)(1-\gamma))B_{1iTS}(t)\right)\\ &- (1/x + \nu + \mu + \mu_{tb})A_{PS}(t)\end{aligned} \tag{8}$$

Additional previously-treated, drug-susceptible active disease cases arise from relapse, and, in DS-TB cases on treatment who have not acquired resistance, they can also result from loss to follow up during effective treatment (probability η) and from loss to follow up or completion treatment failure without immediate retreatment in those on ineffective treatment.

$$\begin{aligned}\frac{dA_{PR}}{dt} &= aE_{PR}(t) + (1/\tau_\omega)W_R(t) + \frac{1}{\tau_{t1}}\sum_T((\delta_1 + (1-\delta_1)(1-\gamma))(\alpha_T B_{1iTS}(t) + B_{1iTR}(t)))\\ &- (1/x + \nu + \mu + \mu_{tb})A_{PR}(t)\end{aligned} \tag{9}$$

Additional previously-treated, drug-resistant active cases (with potential for future effective treatment, and opposed to chronic drug-resistant cases) arise from relapse after acquisition of new resistance during otherwise-successful treatment, or from loss to follow up or completion of ineffective treatment without immediate retreatment in those who are failing treatment with preexisting or newly-acquired resistance, .

Active MDR-TB following unsuccessful treatment attempt

$$\begin{aligned}\frac{dC_R}{dt} &= b\sum_T\left(\frac{s_T}{x}A_{TR}(t) + \frac{s_P}{\tau_{t1}}(B_{1iTR}(t) + \alpha B_{1iTS}(t))\right)\\ &+ \frac{1}{\tau_{t21}}(B_{2i}(t) + B_{2i'}(t) + \delta_2\eta_{\delta 2}B_{2e1}(t) + \delta_2'\eta_{\delta 2}B_{2e1'}(t)) + 1/\tau_\omega V_R(t)\\ &- (\nu + \mu + \mu_{tb})C_R(t)\end{aligned} \tag{10}$$

We assume that individuals who are diagnosed with MDR-TB (from the active state or at the end of unsuccessful DS-TB treatment) but do not initiate treatment,
who fail MDR-TB treatment (due to non-response or loss to follow up), or who relapse after having previously received MDR-TB treatment,
are not retreated for MDR-TB but instead move to a chronic active disease compartment where they remain until spontaneous cure or death.

Treatment states

$$\frac{B_{1eNS}}{dt} = \sigma_{1N}(1/x)A_{NS}(t) - (\mu + 1/\tau_{t1})B_{1eNS}(t) \tag{11}$$

As active drug-susceptible cases are diagnosed and start treatment at rate x, a fraction σ experience initial bacteriologic response to treatment and enter the effective first-line treatment (B_{1e}) states.
Individuals may leave this state due to spontaneous resolution or completion of the treatment course.

$$\frac{dB_{1ePS}}{dt} = \sigma_{1P}\left[(1/x)A_{PS}(t) + 1/\tau_{t1}\sum_T(1-\alpha_T)(1-\delta_1)\gamma B_{1iTS}(t)\right] - (\mu + 1/\tau_{t1})B_{1ePS}(t) \tag{12}$$

For individuals with history of TB-treatment, effective retreatment may be started with probability σ_T after diagnosis of active disease (which may have resulted from failure, relapse, or reinfection) or, in those who remain in care, from immediate retreatment at the end of the ineffective treatment course. Previously-treated patients without MDR have higher probabilities than treatment-naive patients of treatment failure ($\sigma_P > \sigma_N$).

$$\frac{dB_{1iNS}}{dt} = (1-\sigma_{1N})(1/x)A_{NS}(t) - (\mu + i_{B_{1i}}\mu_{tb} + 1/\tau_{t1})B_{1iNS}(t) \tag{13}$$

$$\frac{dB_{1iPS}}{dt} = (1-\sigma_{1P})\left[\frac{1}{x}A_{PS}(t) + \frac{1}{\tau_{t1}}\sum_T (1-\alpha_T)(1-\delta_1)\gamma B_{1iTS}(t)\right] - (\mu + i_{B_{1i}}\mu_{tb} + 1/\tau_{t1})B_{1iPS}(t) \tag{14}$$

$$\frac{dB_{1iNR}}{dt} = (1/x)(1-s_N)A_{NR}(t) - (\mu + i_{B_{1i}}\mu_{tb} + 1/\tau_{t1})B_{1iNR}(t) \tag{15}$$

$$\begin{aligned}\frac{dB_{1iPR}}{dt} &= (1/x)(1-s_P)A_{PR}(t) \\ &+ \frac{1}{\tau_{t1}}\sum_T [(1-\delta_1)(1-s_T)\gamma\left(\alpha_T B_{1iTS}(t) + B_{1iTR}(t)\right)] \\ &- (\mu + i_{B_{1i}}\mu_{tb} + 1/\tau_{t1})B_{1iPR}(t)\end{aligned} \tag{16}$$

For the other $1-\sigma$ fraction of DS-TB patients starting treatment for the first time or being retreated after re-diagnosis or treatment failure, and for all MDR-TB who are given DS-TB treatment despite their MDR disease, first-line treatment is ineffective. This ineffective-treatment state is associated with an added TB mortality rate, which is lower than that of untreated active TB.

$$\begin{aligned}\frac{dB_{2e1}}{dt} &= (1-b)(1-c+cj(1-z))\sigma_2 \sum_T \left[s_T\frac{1}{x}A_{TR}(t) + \frac{s_T}{\tau_{t1}}(1-\delta_1)\gamma(\alpha_T B_{1iTS}(t) + B_{1iTR}(t))\right] \\ &- (\mu + i_{B_{2e1}}\mu_{tb} + \frac{1}{\tau_{t21}})B_{2e1}(t)\end{aligned} \tag{17}$$

$$\begin{aligned}\frac{dB_{2e1'}}{dt} &= (1-b)c(1-j)\sigma_2' \sum_T \left[s_T\frac{1}{x}A_{TR}(t) + \frac{s_T}{\tau_{t1}}(1-\delta_1)\gamma(\alpha_T B_{1iTS}(t) + B_{1iTR}(t))\right] \\ &- (\mu + i_{B_{2e1}}\mu_{tb} + \frac{1}{\tau_{t21}})B_{2e1'}(t)\end{aligned} \tag{18}$$

Of MDR-TB patients who are appropriately diagnosed with drug resistance when initiating treatment or retreatment, a fraction $1-b$ will initiate MDR therapy, and of those, c will be considered for the short regimen, and fraction j of those considered will have disqualifying resistance and require alternative therapy. Of those who start [conventional or short-course] treatment , a fraction [σ_2 or σ_2', respectively] will respond to treatment.
If $z = 0$ (reflecting good outcomes in those ineligible for the short-course regimen), the fraction j who are excluded from the short-course regimen will also have outcomes σ_2, whereas if $z = 1$, they will go to B_{2i} with resulting poor outcomes.

$$\frac{dB_{2e2}}{dt} = (1-\delta_2)\frac{1}{\tau_{t21}}B_{2e1}(t) - (\mu + i_{B_{2e2}}\mu_{tb} + \frac{1}{\tau_{t22}})B_{2e2}(t) \tag{19}$$

$$\frac{dB_{2e2'}}{dt} = (1-\delta_2')\frac{1}{\tau_{t21}}B_{2e1'}(t) - (\mu + i_{B_{2e2'}}\mu_{tb} + \frac{1}{\tau_{t22}'})B_{2e2'}(t) \tag{20}$$

Those who respond to MDR treatment may be lost to follow up at time τ_{t21}, but those not lost to follow up proceed to the latter phase of therapy, which they complete after an additional time τ_{t22} or (for the short regimen) τ_{t22}'.

$$\begin{aligned}\frac{dB_{2i}}{dt} &= (1-b)\left[(1-c+cj(1-z))(1-\sigma_2) + cjz\right]\sum_T \left[s_T\frac{1}{x}A_{TR}(t) + \frac{s_T}{\tau_{t1}}(1-\delta_1)\gamma(\alpha_T B_{1iTS}(t) + B_{1iTR}(t))\right] \\ &- (\mu + i_{B_{2i}}\mu_{tb} + \frac{1}{\tau_{t21}})B_{2i}(t)\end{aligned} \tag{21}$$

$$\begin{aligned}\frac{dB_{2i'}}{dt} &= (1-b)c(1-j)(1-\sigma_2')\sum_T \left[s_T\frac{1}{x}A_{TR}(t) + \frac{s_T}{\tau_{t1}}(1-\delta_1)\gamma(\alpha_T B_{1iTS}(t) + B_{1iTR}(t))\right] \\ &- (\mu + i_{B_{2i}}\mu_{tb} + \frac{1}{\tau_{t21}})B_{2i'}(t)\end{aligned} \tag{22}$$

The remainder of MDR patients (including all of the fraction j who have additional drug resistance, when the short course regimen is being used ($c = 1$) and outcomes of additional resistance are assumed to be poor ($z = 1$)) do not respond to attempts at MDR treatment, and will subsequently move to chronic active disease after time τ_{t21}.

States pending relapse

$$\frac{dW_S}{dt} = \sum_T \frac{\omega_T}{\tau_{t1}}(1-\alpha_T)B_{1eTS}(t) - (\mu + \frac{1}{\tau_\omega})W_S(t) \tag{23}$$

$$\frac{dW_R}{dt} = \sum_T \frac{1}{\tau_{t1}}(\alpha_T)B_{1eTS}(t) - (\mu + \frac{1}{\tau_\omega})W_R(t) \tag{24}$$

$$\frac{dV_R}{dt} = \frac{\omega_2}{\tau_{t22}}B_{2e2}(t) + \frac{\omega_2'}{\tau_{t22}'}B_{2e2'}(t) - (\mu + \frac{1}{\tau_\omega})V_R(t) \tag{25}$$

A specified fraction of individuals who successfully complete treatment will relapse to active disease after a time τ_ω unless they die first. Those drug-resistant relapses that occur after first line treatment (W_S if they did not acquire resistance; W_R if they did) will be eligible for future effective MDR treatment, but because MDr-TB treatment is modeled as representing a full attempt at treatment including any regimen changes that are made mid-course, those relapsing after attempted MDR treatment (V_R) are modeled as not being retreated.

Modeled scenarios

Assumptions about the conventional-treatment baseline projection are the following:

- $s_N = 0.038$; $s_P = 0.67$
- σ_2, ω_2, δ_2 sampled from the specified logit-normal distributions
- $\tau_{t21} = 6/12$; $\tau_{t22} = 14/12$
- $c = 0$

And assumptions in the primary short-course regimen scenario to which it is compared in the primary analysis are the following:

- $s_N = 2 * 0.038 + (2 * 0.67 - 1) * \frac{\text{\# retreatment notifications with MDR}}{\text{\# new notifications with MDR}}$; $s_P = 1$
  (calculated so as to double total MDR-TB diagnoses at time of short-course regimen introduction)
- $\sigma_{2'} = 0.925$; $\omega_{2'} = 0.01$; $\delta_{2'} = \delta_2/2$
- $\tau_{t22'} = 4/12$
- $c = 1$
- j is sampled from the distribution specified in Table 1.
- $z = 1$

The alternative assumptions are specified as the following:

1. $s_N = 0.038$ and $s_P = 0.67$ as in baseline scenario.
2. $\sigma_{2'} = \sigma_2$; $\omega_{2'} = \omega_2$; $z = 0$.
3. $\sigma_{2'} = \sigma_2$; $\omega_{2'} = \omega_2$; $z = 1$.
4. $z = 0$.
5. The originally-sampled distribution for j is multiplied by 3.
6. $j = 0$.
7. $\omega_{2'} = 2\omega_2$.
8. $\delta_{2'} = \delta_2$.

Table S1. Sampled prior distributions for all model parameters

Parameter	Representation in equations above	Median Estimate	Distribution[1]	Sampling Range[2]	References/Notes
Sampled overall model parameters					
Baseline mortality rate (15+ year olds) [$year^{-1}$]	μ	0.0167	Log-normal	0.0155-0.0180	1, using India's life expectancy at age 15
Added mortality rate of untreated symptomatic TB [$year^{-1}$]	μ_{tb}	0.15	Log-normal	0.08 – 0.30	2,3
Probability of rapid progression after initial TB infection	ρ	0.14	Logit-normal	0.08-0.25	4
Protection by latent infection: reduction in rapid progression after second infection event	λ	0.5	Logit-normal	0.1-0.9	4,5
Reactivation rate, latent to early (asymptomatic) active TB [$year^{-1}$]	r	0.001	Log-normal	0.0005-0.002	5–8
Duration of asymptomatic (preclinical) TB if no death or spontaneous resolution [years]	a	0.6	Log-normal	0.36-99	9,10
Infectiousness and mortality of asymptomatic (preclinical) TB, relative to symptomatic TB	i_E	0.22	Logit-normal	0.11-0.40	11,12
Rate of TB diagnosis and treatment initiation (incorporates some pretreatment loss to follow up) [$year^{-1}$]	x	1	Log-normal	0.7-1.5	3,9,13
Rate of spontaneous resolution of untreated active TB [$year^{-1}$]	υ	0.13	Log-normal	0.09-0.20	2
First-line treatment efficacy in DS-TB, new patients[3]	σ_s	0.98	Logit-normal	0.96-0.99	3,14–16
Reduction in first-line treatment efficacy, previously treated DS-TB patients[4]	σ_p/σ_s	0.95	Uniform	0.9-1	3,17
Proportion who relapse, among new DS-TB patients with apparent treatment response	ω_{1n}	0.040	Logit-normal	0.026-0.060	18,19
Increase in relapse risk after first-line therapy, retreatment DS-TB patients[3]	ω_{1p}/ω_{1n}	2	Uniform	1-3	17,18
Median time to relapse, among patients who will relapse [years]	τ_ω	1.5	Log-normal	0.9-2.5	18
Probability of loss to follow up during first-line therapy	δ_1	0.06	Logit-normal	0.03 – 0.1	3
Probability of returning to active TB after loss to follow up during treatment of DS-TB	$\eta_{\delta 1}$	0.4	Logit-normal	0.16-0.7	20,21
Probability of immediate retreatment after DS-TB treatment failure	Γ	0.75	Uniform	0.5-1	Model assumption
Infectiousness and mortality of TB on ineffective treatment, relative to untreated active TB	i_{Bi}	0.5	Uniform	0-1	Model assumption
Sampled drug resistance-related parameters					
Transmissibility of drug-resistant strain, relative to drug-susceptible strain in year 1999	f_R	0.60	Log-normal	0.38-0.94	22–24

Relative rate of decline in TB transmission coefficient, MDR vs DR-TB	e	0.5	Uniform	0-1	Model assumption
Risk of acquired multidrug-resistance during first-line therapy, new DS-TB patients	α_n	0.005	Logit-normal	0.0025-0.01	19
Increase in risk of acquired multidrug-resistance, retreatment DS-TB patients[4]	α_p/α_n	2	Uniform	1-3	17,25
Excess pre-treatment loss to follow up or treatment unavailability after MDR-TB diagnosis	b	0.05	Logit-normal	0.03-0.10	3,13
Treatment efficacy, longer MDR-TB therapy[3]	σ_2	0.77	Logit-normal	0.66-0.85	26–28
Infectiousness during the first six months of effective MDR-TB therapy, relative to untreated active TB	i_{B2e1}	0.1	Uniform	0-0.2	27,29
Relapse risk after finishing apparently-effective longer MDR-TB therapy	ω_2	0.040	Logit-normal	0.015-0.100	27,30
Probability of loss to follow up during longer MDR-TB therapy	δ_2	0.19	Logit-normal	0.14-0.25	3,26,31
Probability of returning to active MDR-TB, after loss to follow up during MDR-TB treatment	$\eta_{\delta 2}$	0.63	Uniform	0.25-1	32,33
Probability of disqualifying resistance for the short-course regimen, among MDR-TB patients with no previous MDR-TB treatment (depends on both resistance prevalence and drug susceptibility assay used)	j	0.1	Logit-normal	0.07-0.15	34
Time since emergence of modern MDR-TB strains [years]		30	Log-normal	20-45	35,36
Parameters specified in calibration or for short-course MDR regimen					
TB transmission coefficient until 1999	β_0	Calibrated to target incidence; see Figure S3			
Rate of decline in TB transmission coefficient, starting in 1999	D	Calibrated to target rate of decline in incidence			
Probability of rifampin susceptibility/MDR testing, new TB patients with MDR-TB	s_N	Increases over time and with short-course regimen			
Probability of rifampin susceptibility/MDR testing, retreatment TB patients with MDR-TB	s_P	Increases over time and with short-course regimen			

[1]Log-normal distributions were used for continuous measures bounded from 0 to infinity, logit-normal distributions for continuous measures bounded from 0 to 1, and uniform distributions when data to suggest a most likely value were missing or sparse.
[2]The ranges shown reflect the 2.5^{th} through 97.5^{th} percentiles of unbounded distributions, or the full range of uniform distributions.
[3]Refers to the fraction of patients with apparent treatment response; includes those who may experience loss to follow up and/or relapse (including, those with who will relapse with acquired resistance)
[4]Modeled differences in treatment outcomes between new and retreatment patients are multiplicative factors and reflect a combination of disease pathology and additional resistance that is not explicitly incorporated into the model.

Table S2: Conversion of parameter values to observed outcomes at the conclusion of treatment (using the median estimates from table S1 and the primary analysis's assumptions about the short-course regimen).

	**Short course**	**Calculation for short-course regimen outcome**	**Conventional therapy**	**Calculation for conventional therapy outcome[2]**
**Respond to treatment:**	**92.5%**	Assumed efficacy	**77%**	Assumed efficacy
Complete, with durable cure	83%	0.925*(1-0.19/2)*(1-0.01)	60%	0.77*(1-0.19)*(1-0.04)
Lost to follow up, with durable cure	3%	0.925*(0.19/2)*(1-0.63)	5%	0.77*(0.19)*(1-0.63)
Complete, with relapse	0.8%	0.925*(1-0.19/2)*(0.01)	2.5%	0.77*(1-0.19)*(0.04)
Lost to follow up, with return to active disease	5.5%	0.925*(0.19/2)*(0.63)	9%	0.77*(0.19)*(0.63)
**Treatment ineffective:**	**7.5%**	1-0.925	**23%**	1-0.77
Death during treatment (death occurs at specified rate during failure and after failure and relapse) [1]	1.7%	(1-0.925)* (1-exp(-(0.15)*20/12))	5%	(1-0.77)*(1-exp(-(0.15)*20/12))
Fail but survive to end of treatment attempt (may die or resolve after treatment)	6.6%	(1-0.925)*exp(-(0.15)*10/12)	18%	(1-0.77)*exp(-(0.15)*20/12)

[1] This, and all other percentages shown, ignore the small constant risk of TB-unrelated death occurring during TB treatment.

[2] These outcomes apply to the overall population of treated MDR-TB patients prior to introduction of the short-course regimen, and to the fraction of patients excluded from the short-course regimen after its introduction (which is 0 in the primary analysis but up to 100% in secondary analyses) who are designated as having "fair outcomes" on conventional therapy. Not show in the table: those who are excluded from the short-course regimen and receive conventional treatment with "poor outcomes" face ongoing rates of both death (~0.15/year) and cure (~0.13/year), such that at two years, 23% have died, 57% persist with active disease, and 20% have resolved; ultimately, 0.15/(0.13+0.15) = 54% die while the other 46% ultimately resolve (through either later, unmodeled treatment attempts or natural mechanisms).

Additional details of model calibration methods:

To capture underlying epidemiologic and biologic uncertainty, we used Latin hypercube sampling to randomly draw sets of model parameters for use in separate simulations. We sampled parameters from log-normal, logit-normal, or, in a few instances of high uncertainty, uniform distributions, estimating medians and ranges from available literature. We sampled parameters in two stages: first we sampled 50,000 sets of parameters relevant to a drug-susceptible TB epidemic and calibrated an epidemic without drug resistance, then we sampled drug-resistance-related parameters (40 nested sets within each DS set) and simulated the emergence of multiple possible drug-resistant TB epidemics within each DS-TB epidemic.

We calibrated each simulated MDR-TB epidemic in a two-stage fashion, by first simulating a drug-susceptible TB epidemic and then adding the historical emergence of MDR within it.[37] Once each drug-susceptible TB epidemic was calibrated at equilibrium to the specified epidemiologic targets, we incorporated the emergence of MDR-TB over time, the current decline in overall TB incidence (modeled via a decline in transmissions per TB case), and the recent expansion of MDR-TB detection. The steps of this calibration process are illustrated in Fig. S1. To replicate the approximately 2% per year decline in DS incidence currently observed in many settings including Southeast Asia [3,38,39], we assumed that the TB transmission coefficient decreased over time (the conservative assumption for the parameter responsible for this decline, when estimating the impact of an intervention intended to prevent transmission). For each sampled set of the other model parameters, we first determined the value of the transmission coefficient that produced a DS-TB epidemic at equilibrium with 30% higher incidence than our year-2014 DS-TB incidence estimate, representing the period before 1999, before the 2% annual decline began. We then determined the linear rate of decline in the transmission coefficient that would, starting from this higher-incidence DS-TB epidemic at equilibrium in 1999, produce the targeted DS-TB incidence (i.e., 98% of the targeted total TB incidence) in 2014.

Figure S1: Stages and timeline of MDR-TB epidemic simulation

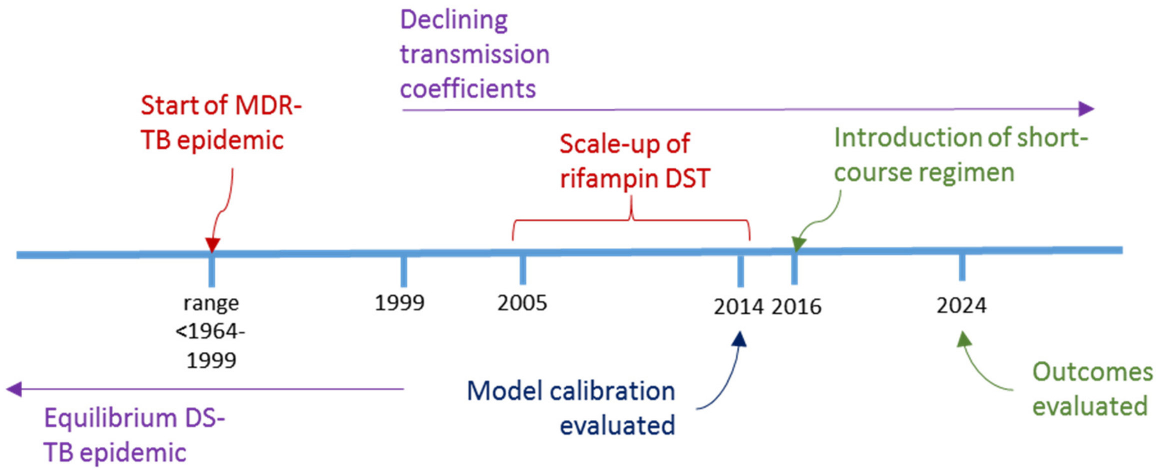

Then, for each DS-TB epidemic and each draw of nested DR-related parameters, we simulated a historical DR-TB epidemic by allowing DR to begin to be acquired during a fraction of DS-TB treatments and then transmitted to others. The effective date when modern DR-TB strains began to emerge and circulate was one of the sampled parameters and was greater than 15 years in the past. As each simulation progressed in time, at 15 years prior to 2014 the transmission coefficient of the DS strain began to decrease at the rate previously determined, and the transmission coefficient of the DR strain began to decrease at a rate that was less rapid by a randomly-chosen factor (to reflect uncertainty in the extent to which factors driving reduced DS TB incidence, including improved socioeconomic and health status, better infection control, and diagnostic and treatment practices, also affect DR TB

in ways not explicitly accounted for in the model). Ten years prior to the present, the use of DST and (longer) MDR treatment began to increase linearly, at separate rates in new versus retreatment patients, to reach reported levels of rifampin DST coverage at the end of 2014. All resulting 2014 epidemics which fell within target ranges (based on WHO-reported confidence intervals) for TB incidence, TB prevalence, TB mortality, MDR prevalence among new TB notifications, and MDR prevalence among retreatment TB notifications, were used to simulate the introduction and impact of the short MDR regimen beginning at the end of 2016.

We converted WHO-reported TB incidence, prevalence, and mortality[3] to the rates specific to adult pulmonary TB by multiplying the reported totals by the fraction of notifications that were pulmonary,[3] multiplying by the estimated incident-dependent fraction of total TB that is estimated to occur in adults,[40] and dividing by the fraction of the Southeast Asian population that is over age 15.[41]

Software: The model was coded and analyses were performed using R 3.2.2[42] and the deSolve package.[43]

Details of rifampin DST coverage assumptions and related sensitivity analysis:

In primary analysis

In our primary baseline scenario, we assumed that rifampin DST coverage (for identification of MDR-TB) remained flat beyond 2014. To achieve a doubling of MDR-TB treatment enrollment when the short-course regimen was introduced, we increased the probability of MDR diagnosis (i.e., drug susceptibility testing) from 67% to 100% in retreatment TB patients and, in new TB patients, from 3.8% to whatever proportion doubles total MDR-TB diagnoses in the first year of use of the short-course regimen. We conservatively assumed that the gap between MDR diagnoses (i.e., notifications) and MDR treatment initiations would persist after introduction of the shorter MDR regimen. The "improved efficacy alone" alternative scenario presented in our primary results assumes that DST coverage remains at 2014 both with and without the short-course regimen.

Sensitivity analyses

In sensitivity analyses shown in this supplement, we considered alternative scenarios in which DST increases over time, regardless of whether a short-course regimen is introduced. In two of these scenarios, we assumed that without the short-course regimen, the same gradual increase in DST use that was modeled from 2004 to 2014 would continue, at the same rates among new and among retreatment patients, until reaching 100%; this led to 100% DST among retreatment patients with five years, but DST coverage remained at only 8% among new patients at the end of the analysis period.

Paralleling the scenarios with and without expanded treatment access in the primary analyses, we paired this alternative baseline of continued gradual DST scale-up with two different assumptions about the impact of the short-course regimen on access to treatment: (1) that use of the short-course regimen increased access to treatment, by increasing DST use at the time of novel regimen introduction in a way that double MDR-TB diagnoses, and then the prior rates of increase in DST use resumed until DST uses reached 100%, and (2) that the expansion of MDR-TB treatment occurred at the same gradual rate only, both with and without the short-course regimen. Results of this sensitivity analysis are shown below.

In a third alternative DST scale-up scenario, we assumed that increasing acceptance of Xpert MTB/Rif led to immediate universalization of rifampin DST among TB cases starting at the present time, regardless of whether the short-course regimen was implemented. Thus, in this scenario there is no possibility for the short-course regimen to further expand access to MDR-TB treatment.

Results of these sensitivity analyses are shown in Figure S4.

Other sensitivity analyses:

Sensitivity to underlying model parameters:

For each sampled parameter (Table 1 and Table S1), we calculated partial rank correlation coefficients (PRCCs) between the parameter value and the corresponding percent reduction in MDR-TB incidence produced by the short-course regimen under our primary assumptions. For those parameters most strongly correlated with the primary outcome (i.e., with absolute value of PRCC>0.2), we then compared the subset of simulations with that parameter in its highest quintile to the subset of simulations with that parameter in its lowest quintile. Results are shown in Figures S5 and S6.

Sensitivity to MDR-TB dynamics

Among all simulations that met calibration targets for the primary setting, and under the assumptions of the primary analysis, we compared the short-course regimen's impact between the subsets of simulations that contained the highest versus lowest quintiles of (1) proportion of MDR-TB incidence due to transmission (rather than acquisition during treatment) and (2) proportion of transmitted MDR-TB due to recent versus remote transmission. Results are shown in Figure S7.

Alternative epidemiologic scenarios:

To evaluate epidemiologic scenarios of higher and lower TB incidence and MDR-TB prevalence, we calibrated the drug-susceptible TB incidence to 500 (higher) or 80 (lower) per 100,000 per year rather than to that of Southeast Asia (200/100,000/year). We allowed the MDR-TB prevalence among new notifications to fall within the lowest fifth (i.e. 0.2-1.3%, "low") or the highest tenth (i.e. 8-34%, "high") of countries with at least 10 new MDR-TB cases in 2014 as estimated by WHO [3], and the MDR-TB prevalence among retreatment notifications to fall within the 20th-80th percentile of countries with a new-notification MDR-TB prevalence in the specified low or high range. Results are shown in Table S3.

Figure S2: Prior and posterior parameter distributions for model parameters that were Latin-hypercube sampled

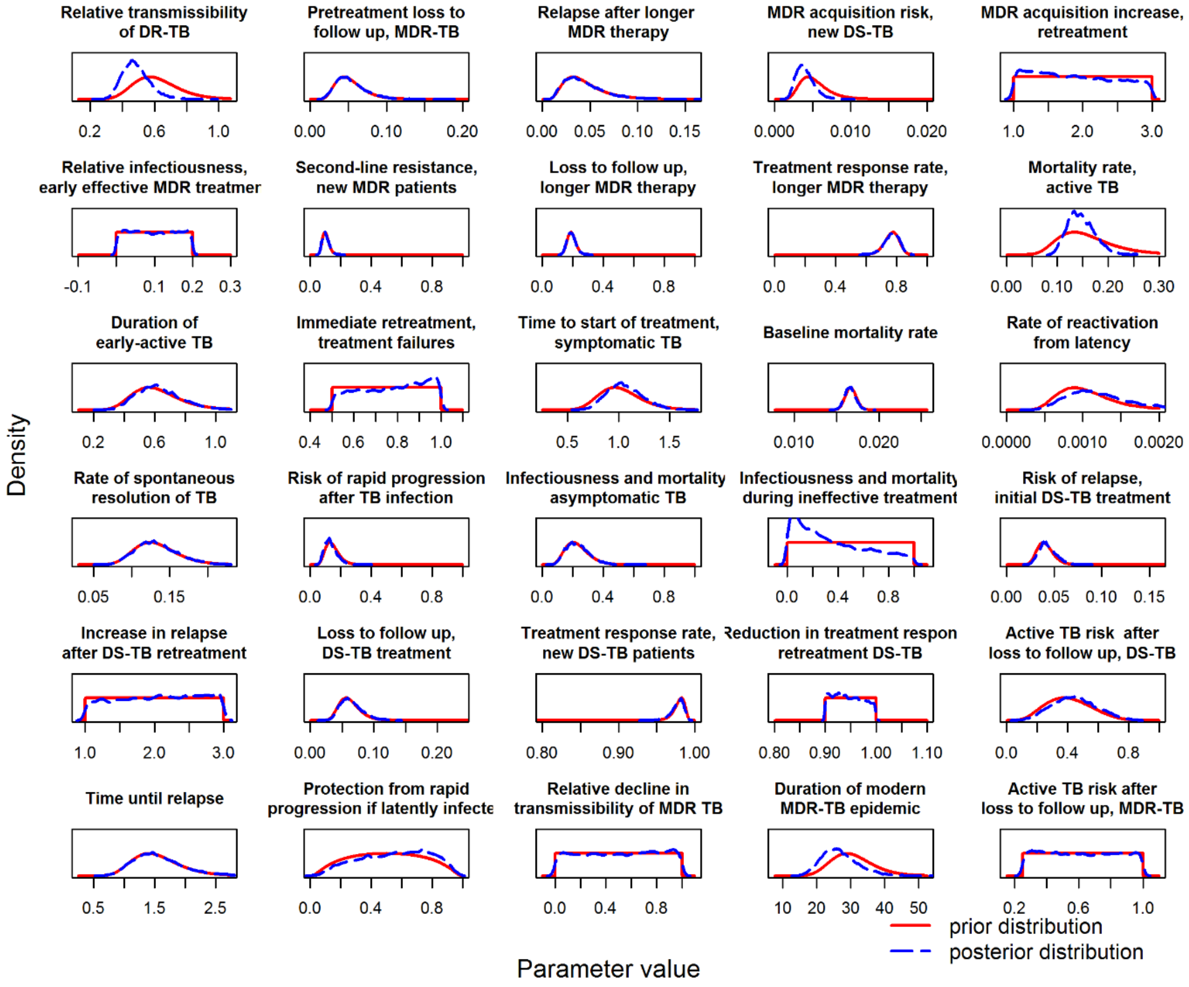

Figure S3: Posterior distribution of the transmission rate for DS and MDR TB, at the time of short-course regimen introduction

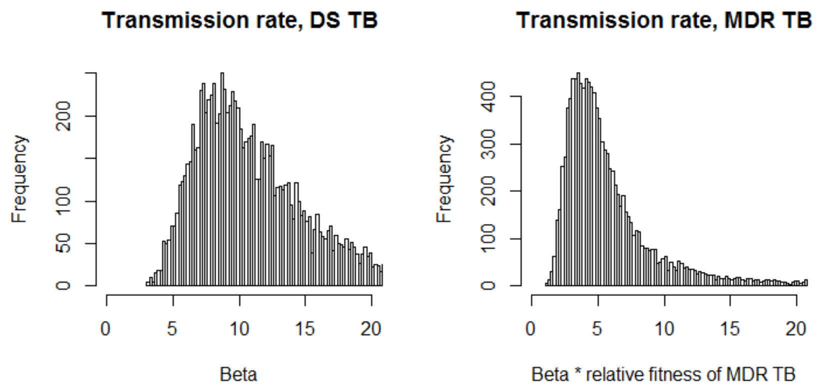

Fig. S4: Sensitivity of short-course regimen impact to assumptions about scale-up of drug susceptibility testing (DST). The top line shows the same primary analysis as in Figure 3: DST beyond 2014 remains flat in the baseline projection and is doubled in a resource-neutral fashion with the short-course regimen. The first alternative (flat DST baseline and no expansion of access with the novel regimen) corresponds to alternative #1 in Figure 3. The additional alternatives compare impact to a baseline of continued gradual DST scale-up, with and without further expansion of DST at the time the short-course regimen is introduced, and to a baseline of immediate implementation of universal DST at time zero (regardless of whether a short-course regimen is introduced).

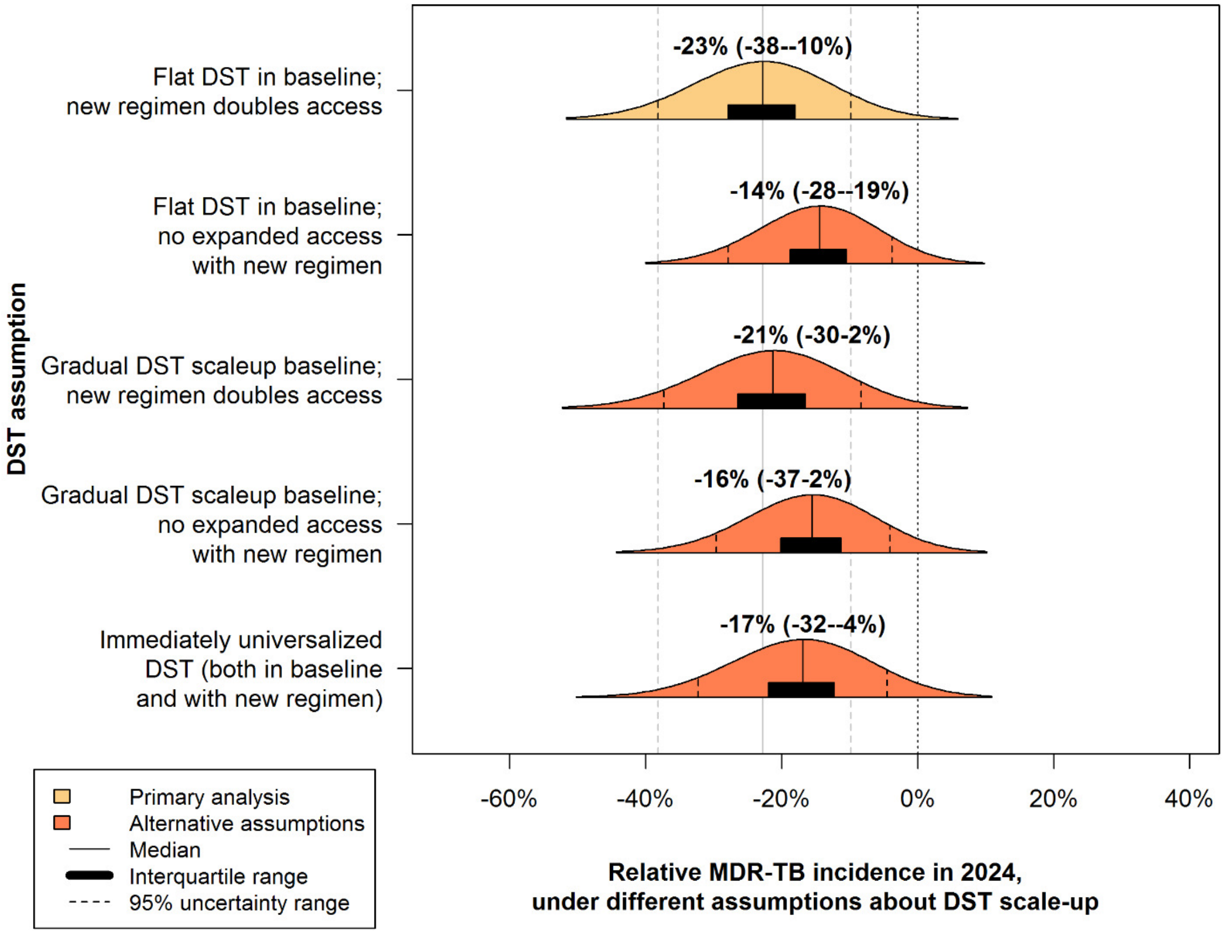

Figure S5: Partial rank correlation coefficients (PRCCs) for model parameters. For each model parameter whose value was chosen by Latin hypercube sampling (LHS) in the calibration process, we calculated PRCCs to determine the association of the parameter with the projected percent reduction in MDR-TB incidence produced by the short-course regimen in the primary scenario, after removing the effects of all other LHS-sampled parameters.

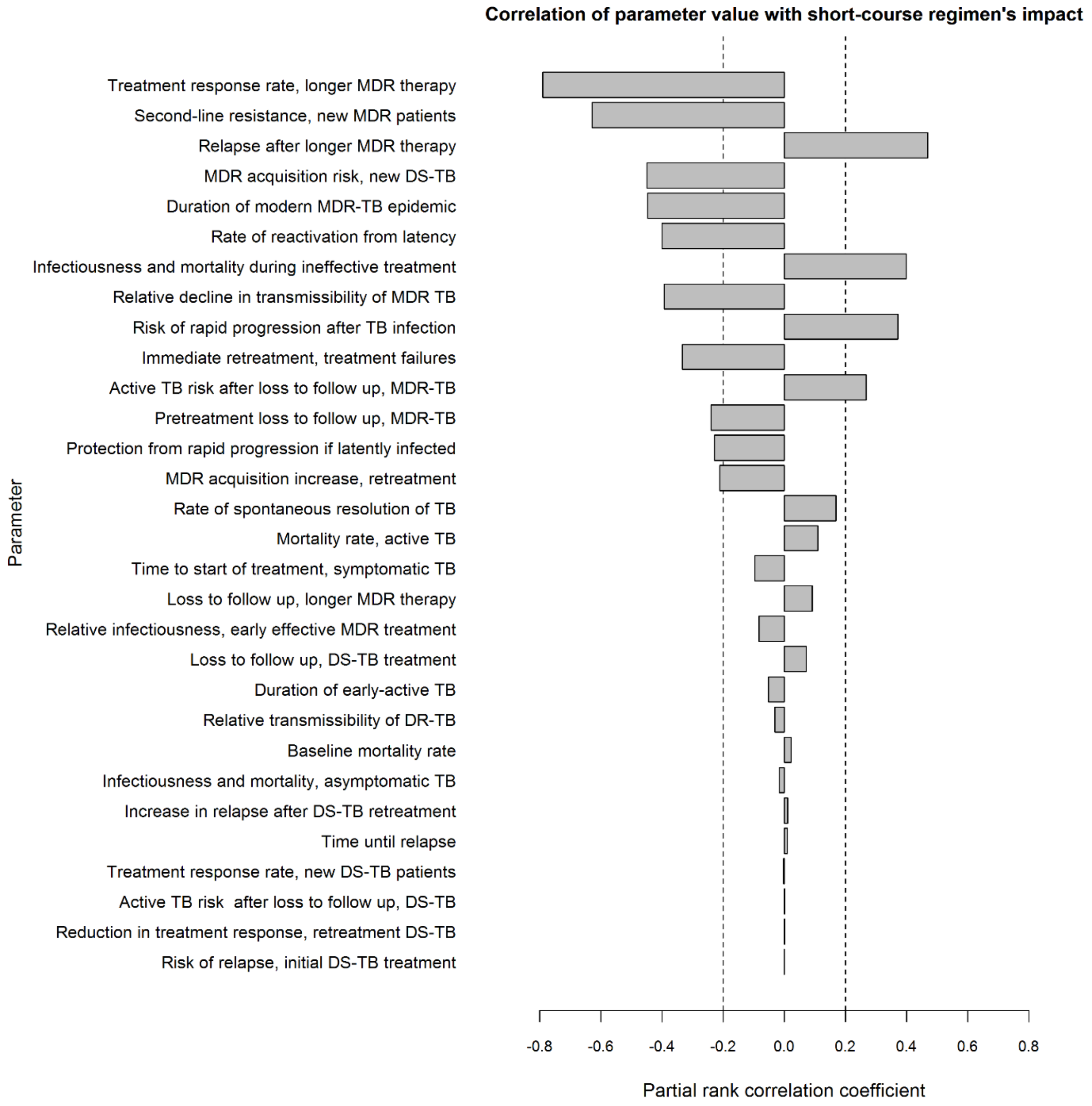

Figure S6: Sensitivity of short-course regimen impact to extremes of most-influential model parameters. For those parameters most strongly correlated with the primary outcome (absolute value of PRCC>0.2), the subset of simulations with that parameter in its highest quintile is compared to the subset of simulations with that parameter in its lowest quintile. Boxes and whiskers show interquartile range (with line at median) and 95% uncertainty range for the short-course regimen's impact.

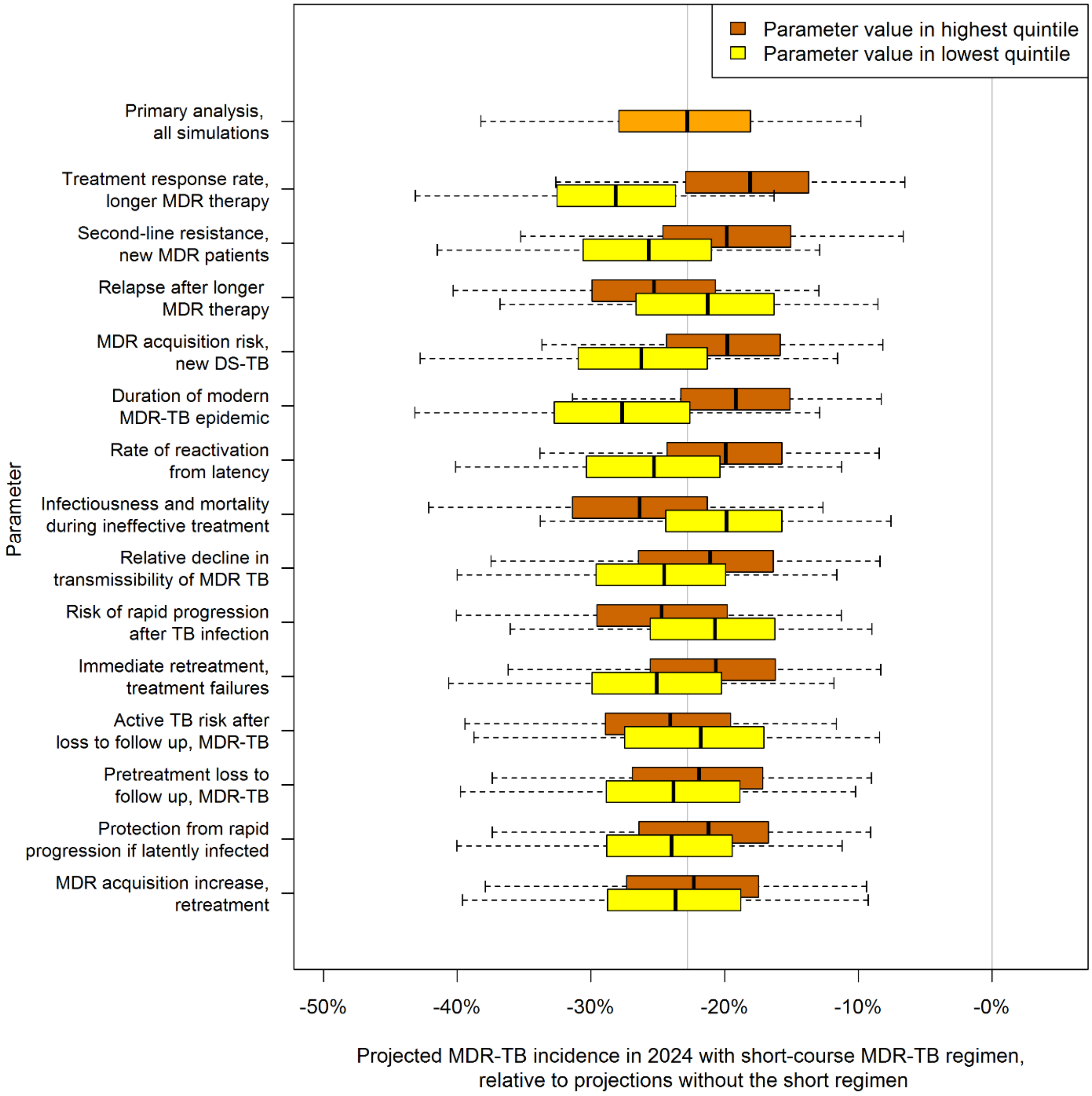

Figure S7: Sensitivity of short-course regimen impact to extremes of MDR-TB acquisition and transmission dynamics. Boxes and whiskers show interquartile range (with line at median) and 95% uncertainty range for the short-course regimen's impact, for the simulations in the highest or lowest quintile of the characteristic indicated.

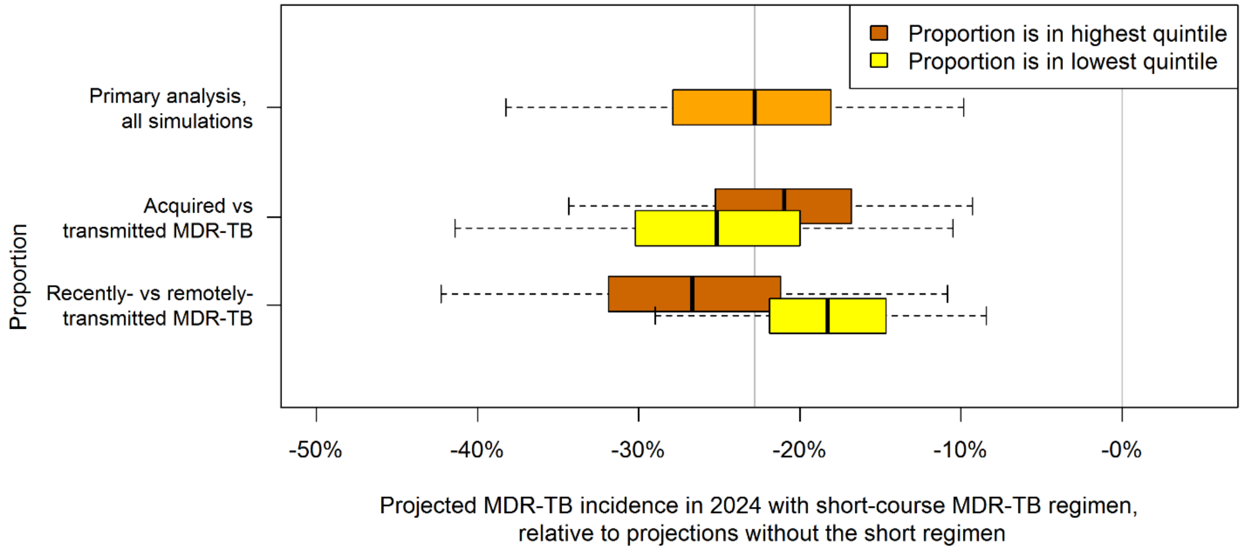

Table S3: Impact of short-course MDR-TB regimen on MDR-TB incidence [median (95% uncertainty range)], under different epidemic scales.

Epidemiol-ogic scenario	TB incidence	MDR-TB preval-ence	Transmitted fraction of MDR-TB incidence, 2016	Recently-transmitted fraction of MDR-TB incidence, 2016	Impact under primary assumptions	Impact with improved efficacy alone	Impact with increased access alone	Impact with more-prevalent disqualifying resistance
**Primary scenario**	Moderate	Moderate	87 (78-93)%	72 (49-85)%	-23% (-38%, -10%)	-15% (-30%, -4%)	-11% (-24%, -3%)	-2% (-20%, +28%)
**Alternative 1**	High	High	98 (95-99)%	78 (56-89)%	-32% (-48%, -16%)	-20% (-37%, -5%)	-19% (-34%, -5%)	-4% (-28%, +34%)
**Alternative 2**	High	Low	76 (59-87)%	61 (41-76)%	-17% (-30%, -7%)	-12% (-23%, -3%)	-8% (-18%, -3%)	-1% (-16%, +23%)
**Alternative 3**	Lower	High	99 (97-99)%	80 (58-90)%	-31% (-46%, -13%)	-17% (-34%, -4%)	-19% (-33%, -7%)	-7% (-27%, +23%)
**Alternative 4**	Lower	Low	74 (56-86)%	53 (32-72)%	-15% (-27%, -6%)	-10% (-21%, -2%)	-7% (-17%, -2%)	-2% (-14%,+16%)

References:

1 Global Health Observatory data repository. World Health Organization http://apps.who.int/gho/data/?theme=main&vid=61830 (accessed Aug 20, 2014).

2 Tiemersma EW, van der Werf MJ, Borgdorff MW, Williams BG, Nagelkerke NJD. Natural History of Tuberculosis: Duration and Fatality of Untreated Pulmonary Tuberculosis in HIV Negative Patients: A Systematic Review. *PLoS One* 2011; **6**: e17601.

3 Global Tuberculosis Report 2015. Geneva: World Health Organization, 2015 http://www.who.int/tb/publications/global_report/en/ (accessed Dec 26, 2015).

4 Vynnycky E, Fine PE. The natural history of tuberculosis: the implications of age-dependent risks of disease and the role of reinfection. *Epidemiol Infect* 1997; **119**: 183–201.

5 Andrews JR, Noubary F, Walensky RP, Cerda R, Losina E, Horsburgh CR. Risk of Progression to Active Tuberculosis Following Reinfection With Mycobacterium tuberculosis. *Clin Infect Dis* 2012; **54**: 784–91.

6 Horsburgh CR, O'Donnell M, Chamblee S, *et al.* Revisiting rates of reactivation tuberculosis: a population-based approach. *Am J Respir Crit Care Med* 2010; **182**: 420–5.

7 Fox GJ, Barry SE, Britton WJ, Marks GB. Contact investigation for tuberculosis: a systematic review and meta-analysis. *Eur Respir J* 2013; **41**: 140–56.

8 Sloot R, Schim van der Loeff MF, Kouw PM, Borgdorff MW. Risk of tuberculosis after recent exposure. A 10-year follow-up study of contacts in Amsterdam. *Am J Respir Crit Care Med* 2014; **190**: 1044–52.

9 Onozaki I, Law I, Sismanidis C, Zignol M, Glaziou P, Floyd K. National tuberculosis prevalence surveys in Asia, 1990–2012: an overview of results and lessons learned. *Trop Med Int Health* 2015; **20**: 1128–45.

10 Dowdy DW, Basu S, Andrews JR. Is passive diagnosis enough? The impact of subclinical disease on diagnostic strategies for tuberculosis. *Am J Respir Crit Care Med* 2013; **187**: 543–51.

11 Behr MA, Warren SA, Salamon H, *et al.* Transmission of Mycobacterium tuberculosis from patients smear-negative for acid-fast bacilli. *Lancet* 1999; **353**: 444–9.

12 Tostmann A, Kik SV, Kalisvaart NA, *et al.* Tuberculosis Transmission by Patients with Smear-Negative Pulmonary Tuberculosis in a Large Cohort in The Netherlands. *Clin Infect Dis* 2008; **47**: 1135–42.

13 MacPherson P, Houben RM, Glynn JR, *et al.* Pre-treatment loss to follow-up in tuberculosis patients in low- and lower-middle-income countries and high-burden countries: a systematic review and meta-analysis. *Bull World Health Organ* 2014; **92**: 126–38.

14 Gillespie SH, Crook AM, McHugh TD, *et al.* Four-month moxifloxacin-based regimens for drug-sensitive tuberculosis. *N Engl J Med* 2014; **371**: 1577–87.

15 Merle CS, Fielding K, Sow OB, *et al.* A four-month gatifloxacin-containing regimen for treating tuberculosis. *N Engl J Med* 2014; **371**: 1588–98.

16 Jindani A, Harrison TS, Nunn AJ, *et al.* High-dose rifapentine with moxifloxacin for pulmonary tuberculosis. *N Engl J Med* 2014; **371**: 1599–608.

17 Menzies D, Benedetti A, Paydar A, *et al.* Standardized treatment of active tuberculosis in patients with previous treatment and/or with mono-resistance to isoniazid: a systematic review and meta-analysis. *PLoS Med* 2009; **6**: e1000150.

18 Marx FM, Dunbar R, Enarson DA, *et al.* The Temporal Dynamics of Relapse and Reinfection Tuberculosis After Successful Treatment: A Retrospective Cohort Study. *Clin Infect Dis* 2014; **58**: 1676–83.

19 Menzies D, Benedetti A, Paydar A, *et al.* Effect of duration and intermittency of rifampin on tuberculosis treatment outcomes: a systematic review and meta-analysis. *PLoS Med* 2009; **6**: e1000146.

20 Kruk ME, Schwalbe NR, Aguiar CA. Timing of default from tuberculosis treatment: a systematic review. *Trop Med Int Health* 2008; **13**: 703–12.

21 Hong Kong Chest Service, Tuberculosis Research Centre Madras, and British Medical Research Council. A controlled trial of 2-month, 3-month, and 12-month regimens of chemotherapy for sputum-smear-negative pulmonary tuberculosis. Results at 60 months. *Am Rev Respir Dis* 1984; **130**: 23–8.

22 Borrell S, Gagneux S. Infectiousness, reproductive fitness and evolution of drug-resistant Mycobacterium tuberculosis [State of the art]. *Int J Tuberc Lung Dis* 2009; **13**: 1456–66.

23 Billington OJ, McHugh TD, Gillespie SH. Physiological Cost of Rifampin Resistance Induced In Vitro in Mycobacterium tuberculosis. *Antimicrob Agents Chemother* 1999; **43**: 1866–9.

24 Grandjean L, Gilman RH, Martin L, *et al.* Transmission of Multidrug-Resistant and Drug-Susceptible Tuberculosis within Households: A Prospective Cohort Study. *PLoS Med* 2015; **12**: e1001843.

25 Lew W, Pai M, Oxlade O, Martin D, Menzies D. Initial drug resistance and tuberculosis treatment outcomes: systematic review and meta-analysis. *Ann Intern Med* 2008; **149**: 123–34.

26 Johnston JC, Shahidi NC, Sadatsafavi M, Fitzgerald JM. Treatment outcomes of multidrug-resistant tuberculosis: a systematic review and meta-analysis. *PLoS One* 2009; **4**: e6914.

27 Holtz TH, Sternberg M, Kammerer S, *et al.* Time to sputum culture conversion in multidrug-resistant tuberculosis: predictors and relationship to treatment outcome. *Ann Intern Med* 2006; **144**: 650–9.

28 Ahuja SD, Ashkin D, Avendano M, *et al.* Multidrug Resistant Pulmonary Tuberculosis Treatment Regimens and Patient Outcomes: An Individual Patient Data Meta-analysis of 9,153 Patients. *PLoS Med* 2012; **9**: e1001300.

29 Dharmadhikari AS, Mphahlele M, Venter K, *et al.* Rapid impact of effective treatment on transmission of multidrug-resistant tuberculosis. *Int J Tuberc Lung Dis* 2014; **18**: 1019–25.

30 Ahmad Khan F, Gelmanova IY, Franke MF, *et al.* Aggressive Regimens Reduce Risk of Recurrence After Successful Treatment of MDR-TB. *Clin Infect Dis Off Publ Infect Dis Soc Am* 2016; published online May 8. DOI:10.1093/cid/ciw276.

31 Toczek A, Cox H, du Cros P, Cooke G, Ford N. Strategies for reducing treatment default in drug-resistant tuberculosis: systematic review and meta-analysis [Review article]. *Int J Tuberc Lung Dis* 2013; **17**: 299–307.

32 Kliiman K, Altraja A. Predictors and mortality associated with treatment default in pulmonary tuberculosis. *Int J Tuberc Lung Dis* 2010; **14**: 454–63.

33 Franke MF, Appleton SC, Bayona J, *et al.* Risk factors and mortality associated with default from multidrug-resistant tuberculosis treatment. *Clin Infect Dis* 2008; **46**: 1844–51.

34 Kurbatova EV, Dalton T, Ershova J, *et al.* Additional drug resistance of multidrug-resistant tuberculosis in patients in 9 countries. *Emerg Infect Dis* 2015; **21**: 977–83.

35 Merker M, Blin C, Mona S, *et al.* Evolutionary history and global spread of the Mycobacterium tuberculosis Beijing lineage. *Nat Genet* 2015; **47**: 242–9.

36 Cohen KA, Abeel T, Manson McGuire A, *et al.* Evolution of Extensively Drug-Resistant Tuberculosis over Four Decades: Whole Genome Sequencing and Dating Analysis of Mycobacterium tuberculosis Isolates from KwaZulu-Natal. *PLoS Med* 2015; **12**: e1001880.

37 Kendall EA, Fofana MO, Dowdy DW. Burden of transmitted multidrug resistance in epidemics of tuberculosis: a transmission modelling analysis. *Lancet Respir Med* 2015; **3**: 963–72.

38 Glaziou P, Falzon D, Floyd K, Raviglione M. Global epidemiology of tuberculosis. *Semin Respir Crit Care Med* 2013; **34**: 3–16.

39 World Health Organization Regional Office for South-East Asia. Tuberculosis control in the South-East Asia Region: Annual TB Report 2015. 2015. http://www.searo.who.int/tb/annual-tb-report-2015.pdf (accessed July 27, 2016).

40 Donald PR. Childhood tuberculosis: out of control? *Curr Opin Pulm Med* 2002; **8**: 178–82.

41 United Nations Department of Economic and Social Affairs. World Population Prospects, the 2015 Revision. 2016 https://esa.un.org/unpd/wpp/ (accessed July 1, 2016).

42 R Core Team. R: A Language and Environment for Statistical Computing. Vienna, Austria: R Foundation for Statistical Computing, 2014 http://www.R-project.org/.

43 Soetaert K, Petzoldt T, Setzer RW. Solving Differential Equations in R: Package deSolve. *J Stat Softw* 2010; **33**: 1–25.
